# Peripheral tissue BDNF expression is affected by promoter IV defect and enriched environments in mice: negative hippocampus-intestine and positive thymus-serum-muscle correlations

**DOI:** 10.1186/s10020-025-01196-4

**Published:** 2025-05-02

**Authors:** Janet Wang, William Schupp, Kazuko Sakata

**Affiliations:** 1 Department of Pharmacology, College of Medicine, University of Tennessee Health Science Center, 71 S. Manassas St. Room 225N, Memphis, TN 38103 USA; 2 Department of Psychiatry, College of Medicine, University of Tennessee Health Science Center, Memphis, USA

**Keywords:** BDNF, Peripheral tissues, Enriched environment treatment, Promoter IV knock-in mice, Inter-organ correlations

## Abstract

**Background:**

Brain-derived neurotrophic factor (BDNF) expression is reduced in the brain of various central nervous system (CNS) disorders, but its relation to peripheral expression remains unclear. This study aimed to determine peripheral BDNF expression affected by BDNF promoter IV defect and enriched environment treatment (EET). Promoter IV defect is associated with CNS disorders and chronic stress, whereas EET increases hippocampal BDNF expression and ameliorates CNS dysfunctions.

**Methods:**

Enzyme-linked immunosorbent assay measured BDNF protein levels in eleven regions (hippocampus, frontal cortex, heart, lung, liver, spleen, intestine, kidney, intestine, thymus, muscle, serum) in wild-type and knock-in promoter IV (KIV) mice with or without 3 weeks of EET provided after weaning.

**Results:**

Knock-in promoter IV resulted in BDNF levels significantly decreased in muscle, but significantly increased in intestine, liver, thymus, and serum, which suggests compensatory upregulation of other promoters in those tissues. EET increased BDNF levels in muscle and serum of KIV mice and thymus of wild-type mice, suggesting EET’s beneficial effects in muscle motor and adaptive immune regulation. EET increased hippocampal BDNF levels in both genotypes, which significantly negatively correlated with intestine BDNF levels, suggesting its role in the brain-gut axis. EET reduced wild-type heart BDNF levels, possibly through parasympathetic regulation. Significant positive BDNF correlations were observed among serum-muscle, serum-thymus, lung-spleen, and intestine-liver, suggesting inter-organ interaction and regulation of BDNF. Partial Least Squares discriminant analyses (PLS-DA) identified that variations in BDNF levels in intestine, liver, frontal cortex, and serum contribute most to classify promoter IV defect, and those in hippocampus, serum, heart, thymus, and liver contribute most to classify EET effects.

**Conclusion:**

This is the first study to demonstrate how genetic and environmental factors affect BDNF expression in peripheral tissues, highlighting the complex BDNF correlations across organ systems and suggesting usefulness of multivariate BDNF analyses for detecting promoter IV defect and enriched environment effects. Elucidation of BDNF’s role and regulatory mechanisms in peripheral organ systems may help better our understanding of its connection to CNS disorders and their treatments.

**Supplementary Information:**

The online version contains supplementary material available at 10.1186/s10020-025-01196-4.

## Introduction

Brain-derived neurotrophic factor (BDNF) is a neuronal growth factor that promotes neuronal functions and plasticity (Thoenen 1995; Lu 2003; Park and Poo 2013). However, its expression is reduced in the brain, particularly in the hippocampus and prefrontal cortex, in various mental disorders, such as major depressive disorder (Dwivedi et al. 2003), schizophrenia (Weickert et al. 2003; Hashimoto et al. 2005), and Alzheimer's disease (Phillips et al. 1991; Connor et al. 1997). BDNF expression is controlled by nine promoters in both humans (Pruunsild et al. 2007) and rodents (Aid et al. 2007). Among the nine promoters, promoter IV (called promoter III before 2007) is the most neuronal activity-responsive promoter (Metsis et al. 1993; Tao et al. 1998; Shieh et al. 1998), but becomes inactivated by chronic stress through epigenetic processes (Tsankova et al. 2006; Roth et al. 2009). Reduced activity of promoter IV is observed and implicated in depression (Keller et al. 2010; Hing et al. 2012), schizophrenia (Wong et al. 2010), and Alzheimer's disease (Aarons et al. 2019; Sen et al. 2015). Blocking promoter IV-driven BDNF expression (pIV-BDNF) in knock-in promoter IV (KIV) mice (Sakata et al. 2009) results in depression-related behaviors, such as decreased explorative activity and increased stress-induced despair, and cognitive inflexibility, such as reduced fear extinction and impaired response inhibition (Sakata et al. 2010; Sakata et al. 2013a). Functionally, lack of pIV-BDNF impairs long-term synaptic plasticity (LTP) at late phase (> 2 h) in the hippocampus (Sakata et al. 2013a), decreases GABAergic functions in the prefrontal cortex (Sakata et al. 2009), and disturbs expression of neurotransmitter-related genes in glutamatergic, GABAergic, monoaminergic, and cholinergic systems (e.g., *GRIN2A/B, GRM5, GABRA5, GABRR1 5HTT, 5HTR1B*, and *CHRNA5*) (Sakata and Duke 2014; Sakata and Overacre 2017; Dong et al. 2020).

To reverse the defects caused by pIV-BDNF deficiency, we previously found that enriched environment treatment (EET), which stimulates mental, physical, and social activity, is more effective than treatments with monoamine-based antidepressant drugs (Jha et al. 2011; Sakata et al. 2013b). EET increases BDNF expression via multiple promoters (I, II, and III) other than IV to compensate for the pIV-BDNF deficiency in the hippocampus, and reverses depression-like behaviors of KIV mice (Jha et al. 2011; Dong et al. 2018). EET also normalizes disturbed expression of neurotransmitter genes in the frontal cortex of KIV mice (Sakata and Overacre 2017; Dong et al. 2020). These effects are larger when EET is provided during early-life development than at aged adulthood (Dong et al. 2020, 2018; Jha et al. 2016).

While numerous studies have focused on the brain to examine BDNF expression levels and EET effects, those in the peripheral organ systems remain unclear. Despite the name of 'brain-derived' neurotrophic factor, the BDNF gene is widely expressed in organs other than the brain (e.g., heart, lung, and muscles (Aid et al. 2007; Lommatzsch et al. 1999; Lommatzsch et al. 2005; Esvald et al. 2023)). In addition, BDNF levels in blood (e.g., serum and plasma) have been extensively examined as a possible biomarker reflecting mental conditions, such as depression (Karege et al. 2002; Sen et al. 2008; Molendijk et al. 2014), schizophrenia (Fernandes et al. 2015), and Alzheimer’s disease (Yasutake et al. 2006), because measuring BDNF levels in the brains of living humans is difficult. We hypothesized that peripheral BDNF expression levels may also be affected by promoter IV defects and EET, correlating with brain BDNF levels. The purpose of this study was to test this hypothesis by determining 1) peripheral distribution of BDNF expression and how it is affected by defective promoter IV, 2) EET effects on peripheral BDNF expression, and 3) correlations between brain BDNF levels and peripheral BDNF levels in mice.

## Method

*Animals*: Wild-type (WT) mice and knock-in BDNF promoter IV (KIV) mice were used. Generation of KIV is described in Sakata et al. (2009). Briefly, a green-fluorescent-protein gene with a stop codon was inserted into exon IV, which disrupts promoter IV-driven expression of BDNF protein. KIV mice were produced from 129/sv embryonic stem cells with C57BL/6 J blastocytes and then crossed to C57BL/6 J females (The Jackson Lab, Bar Harbor, ME) over 12 generations. Heterozygous mice were bred to produce WT and KIV littermates of the same genetic background. Offspring from these littermates were used. All animals were group-housed in a climate-controlled vivarium in a normal 12:12 h-dark–light cycle with food and water ad libitum. All animal experiments were approved by the University of Tennessee Laboratory Animal Care and Use Committee (#20–0188.0) and followed the ARRIVE (Animal Research Reporting of In Vivo Experiments) guidelines.

*Treatment:* A total of 48 mice, consisting of 24 WT and 24 KIV mice, were used to collect data from N = 12 per group for 2 treatment conditions: standard condition treatment (SCT) and EET. Equal numbers of males (N = 6) and females (N = 6) were used per group. Mice at 3–4 weeks of weaning age were randomly placed in either SCT or EET for 3 weeks. The same treatment conditions that normalized depression-like behavior of KIV mice were used (Jha et al. 2011; Jha et al. 2016; Dong et al. 2018). Briefly, SCT is defined by a regular cage (27 × 16 × 12 cm) containing 2–5 mice to avoid isolation stress. EET consisted of a large cage (44 × 22 × 16 cm) that contained two plastic running wheels to increase physical exercise, toys (igloos, tunnels, balls, etc.) to increase cognitive activity, and 5–10 mice to increase social interaction. Additionally, in the EET condition, toys were changed weekly, and a teaspoon of rodent foraging crumble (#5783, Bio-Serv, NJ) was sprinkled across the cage weekly to increase exploratory activity of animals.

*Tissue Processing*: After the treatments, all mice were given isoflurane (1–3 ml/L vapor) for rapid anesthesia and euthanized by cardio-puncture blood withdrawal (0.5–1 mL) and decapitation. The blood samples were processed to obtain serum as described previously (Karege et al. 2002). Samples of hippocampus, frontal cortex (FC), heart, lung, liver, spleen, intestine, kidneys, thymus, and leg muscle were collected and immediately frozen on dry ice. Samples were kept in a -80 °C freezer until processed. Tissues were collected between 14:00 and 18:00 to minimize diurnal effects on BDNF expression (Berchtold et al. 1999).

*BDNF Enzyme-linked immunosorbent assay (ELISA)*: Ice-cold lysis buffer (20 mM Tris, pH 7.5; 150 mM NaCl; 1% Nonidet P-40; 1 mM EDTA) was prepared with freshly added protease inhibitors (10 μg/mL aprotinin, 1 μg/mL leupeptin, 1 μg/mL pepstatin, and 1 mM PMSF). Brain tissues in 300 μL of lysis buffer were homogenized by pipetting and sonication (Branson Fisher: pulse with 3 s on and 1 s off × 8 cycles at 30% power). Peripheral organ tissues were homogenized using TissueLyser II (Qiagen): 30 mg of each tissue sample was added 300 μL of lysis buffer and one bead (#69,989, Qiagen) in a 2 mL round bottom tube (#022363352, Eppendorf), and then were homogenized for 3 min at 25 Hz. The samples were kept on ice for 30 min for additional cell lysis and then were centrifuged for 5 min at 13,000 × g at 4 °C. Supernatants were collected. For each supernatant sample, BDNF concentration was measured by ELISA (#G7611, Promega; DY248, R&D systems), and total soluble protein level was measured by DC™ protein assays (#500–0002, Bio-Rad Laboratories), as described by the manufactures. BDNF signal was normalized to the total soluble protein level in each sample (pg/mg total protein). Then, the relative BDNF concentration levels were calculated for each animal: % hippocampus for examining tissue distribution, % WT for examining genotype effects, % SCT for examining EET effects.

*Correlation studies*: Program R (RStudio, Posit Software, PBC) was used for Pearson’s correlation analyses, partial-least squares (PLS) regression analyses, and PLS discriminant analysis (PLS-DA) with variable importance to the projection (VIP) (Rohart et al. 2017), and statistics. Data of BDNF levels of 11 regions that were measured on the same ELISA plate were used to avoid variations caused by different plates and experimental handling (N = 8 mice from N = 2 mice per group × 4 groups per plate × 3 plates: N = 24 mice).

*Statistical Analyses:* Student’s *t*-tests were performed to compare two data groups. Two-way analyses of variance (ANOVA) were performed using PRISM software (GraphPad) to examine genotype and treatment effects, and, when warranted, post hoc Bonferroni multiple comparisons were performed. Outlier data defined as 2 standard deviations above or below the mean were excluded because they likely resulted from technical errors. Otherwise, no exclusion criteria were pre-determined. The Jarque–Bera normality tests were performed to analyze the data distribution for each group. All data were normally distributed (*p* > 0.05). The sample size was decided based on previous experiments and power analyses (Dong et al. 2018). Statistical significance was set at *p < 0.05. Data are presented as means and standard error of the mean. Statistical results are presented in Supplementary Table 1.

## Results

### Distribution of BDNF expression

**Fig. 1:**
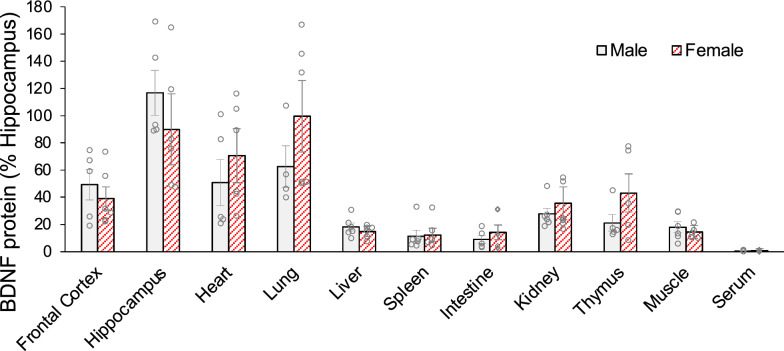
Tissue distribution of BDNF in WT mice in the standard condition treatment. Note the BDNF levels are highest in the hippocampus, moderate in other organs, and lowest in the serum. No significant differences between males and females were observed in any brain and peripheral regions (*p* > 0.05). N = 6 mice/group. BDNF protein levels (pg/mg total protein) were normalized to the hippocampal BDNF levels (%) and are shown as means ± SE

First, we examined BDNF distribution in WT mice with the standard condition treatment (SCT). BDNF concentrations were highest in the hippocampus (Table 1). The distribution of BDNF levels was examined as % hippocampus BDNF concentration to compare peripheral levels relative to the hippocampal levels (Fig. 1). Peripherally, the highest levels were found in the lung. Moderate levels of BDNF were observed in the frontal cortex (FC), heart, kidney, and thymus, with the lowest levels of BDNF concentrations in serum. There were no significant differences between males and females in BDNF concentrations in any regions (*p* > 0.05, Student’s *t*-test).

**Table 1:** The BDNF concentration in WT mice in the standard condition treatment (pg/mg total protein)

	FC	Hip	Heart	Lung	Liver	Spleen	Int	Kidney	Thy	Mus	Serum
Mean	371	636	230	390	118	43	34	276	149	80	1.05
SE	108	172	45	62	35	8	5	88	40	18	0.09
SD	371	596	156	215	121	28	17	304	137	62	0.3
Min	19	48	84	108	8	9	13	10	13	14	0.5
Max	875	1672	519	666	360	90	70	764	523	188	1.6

*FC* frontal cortex, *Hip* hippocampus, *Int* intestine, *Thy* thymus, *Mus* muscle

### Effects of promoter IV defect on peripheral BDNF expression

**Fig. 2:**
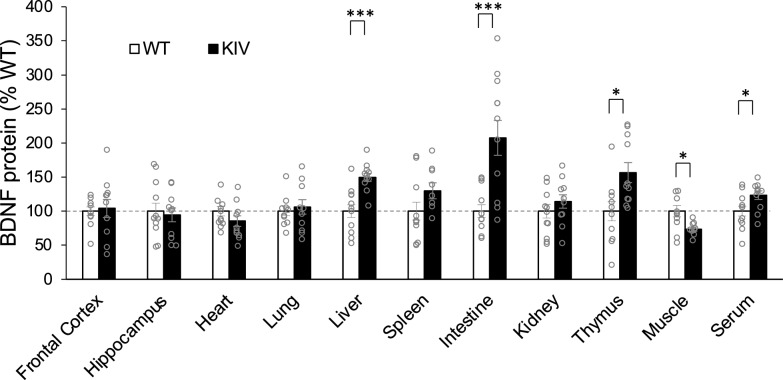
Defective promoter IV affected peripheral BDNF expression in the standard condition treatment. KIV mice showed significantly higher BDNF levels in the liver, intestine, thymus, and serum, but lower BDNF levels in the muscle compared to WT mice. *p < 0.05; ***p < 0.005; by Student *t*-test. N = 12 mice/group. BDNF protein levels (pg/mg total protein) were normalized to the mean BDNF levels in each tissue of WT mice (%) and are shown as means ± SE

Next, we examined the effects of lack of pIV-BDNF in KIV mice. KIV mice with SCT, compared to their WT counterparts (WT-SCT), showed significantly reduced BDNF levels in the muscle, but significantly increased BDNF levels in the liver, intestine, thymus, and serum (at least *p* < 0.05 for each, *t*-test, Fig. 2). There were no sex differences in any tissues of KIV mice (*p* > 0.05), while both males and female KIV mice showed similar BDNF expression changes compared to WT mice (Supplementary Fig. 1).

### Effects of enriched environment treatment (EET) on peripheral BDNF expression

**Fig. 3:**
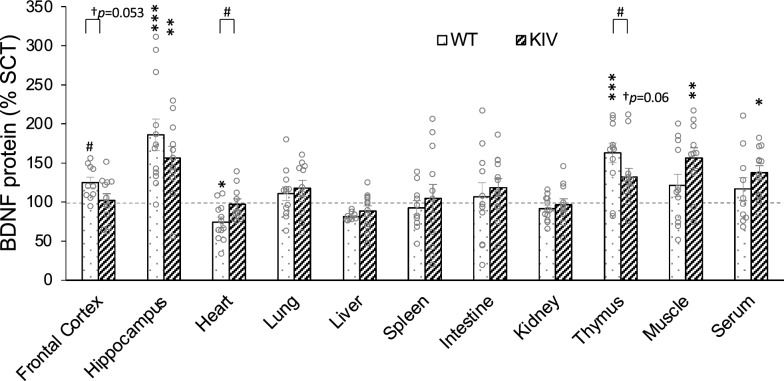
Enriched environment treatment (EET) effects on BDNF expression. EET significantly increased BDNF levels in the hippocampus of both WT and KIV mice, thymus of WT mice, and muscle and serum of KIV mice, and significantly reduced BDNF levels in the heart of WT mice. EET vs. SCT: *p < 0.05; **p < 0.05; ***p < 0.005 by Bonferroni post hoc test. By Student *t*-test, EET showed increased BDNF levels in the WT frontal cortex (^#^*p* < 0.05 by *t*-test), while showing a trend of increase in the KIV thymus (^†^*p* = 0.060). No significant differences in EET effects between genotypes in any regions were observed by Bonferroni post hoc test (WT vs KIV: *p* > 0.05), while genotype differences were observed in the heart, thymus, and frontal cortex by *t*-test (^#^*p* < 0.05 or ^†^*p* = 0.053). N = 12 mice/group. BDNF levels (pg/mg total protein) were normalized to the mean BDNF levels of each genotype with standard control treatment (% SCT). Data are shown as means ± SE

Further, we examined EET effects by comparing EET and SCT groups. EET significantly increased BDNF levels in the hippocampus of both WT and KIV mice (EET vs. SCT: *p* < 0.05 for WT or KIV, Two-way ANOVA with Bonferroni’s comparison, Fig. 3), reproducing previous results (Jha et al. 2011, 2016) and assuring proper experimental procedures in this study. Peripherally, EET significantly increased BDNF levels in the thymus of WT mice and in the muscle and serum of KIV mice (at least *p* < 0.05 by Bonferroni’s comparison, Fig. 3). EET also showed a trend of increasing BDNF levels in the KIV thymus (KIV-EET vs KIV-SCT: *p* = 0.06 by *t*-test) and WT frontal cortex (WT-EET vs WT-SCT: *p* = 0.01 by *t*-test, Supplementary Table 1_Tab 3). EET significantly reduced BDNF levels in the heart of WT mice (at least *p* < 0.05 by Bonferroni’s comparison, Fig. 3). EET did not change BDNF levels in other regions.

No genotype difference in EET effects was observed for any regions (WT vs. KIV: *p* > 0.05) by two-way ANOVA with Bonferroni’s comparison, although *t*-test showed significant genotype difference and its trend in the heart, thymus, and frontal cortex (^#^*p* ≤ 0.05 by *t*-test, Fig. 3). No significant sex effects were observed in the EET effects (males vs. females: *p* > 0.05 by *t*-test, Supplementary Fig. 2a) except that EET-induced BDNF upregulation was larger in females than in males in the hippocampus of WT mice (*p* < 0.05 by *t*-test, Supplementary Fig. 2b).

### Correlation in BDNF levels across regions.

**Fig. 4:**
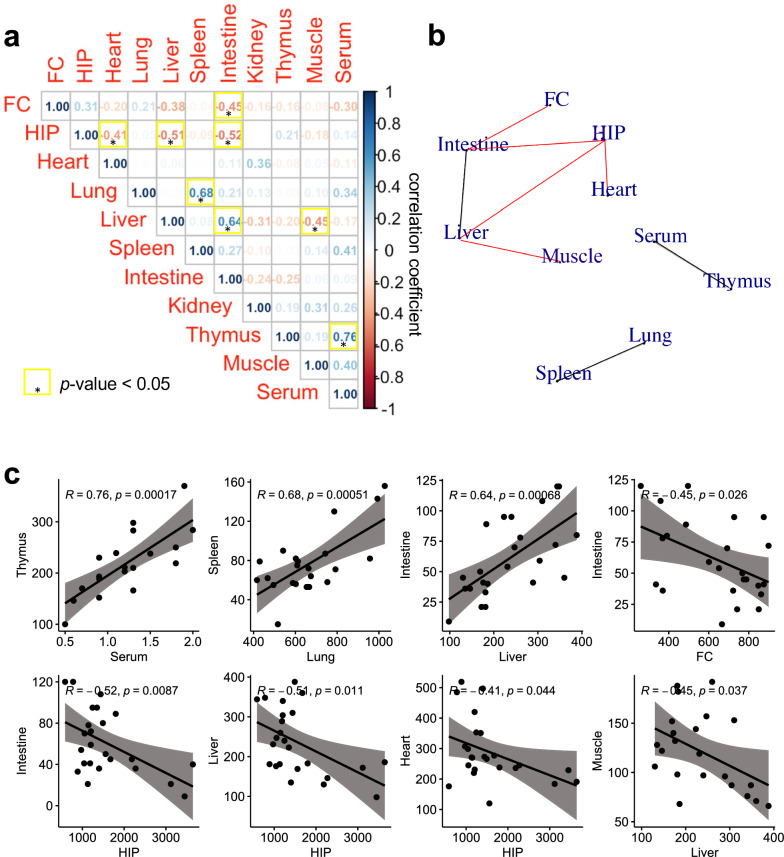
Correlation of BDNF levels across regions. a. Pearson's correlation analyses were conducted for BDNF levels from all groups including WT and KIV mice with SCT and EET. The correlation coefficient (r) with significance (*p < 0.05) is highlighted. b. Significant positive or negative correlations are connected by lines in black or red, respectively. c. BDNF protein levels (pg/mg total protein) are plotted by comparing two regions. N = 24 mice. HIP: hippocampus; FC: frontal cortex

Finally, we examined how BDNF levels correlate among different regions. Correlation analyses using all four groups (WT-SCT, KIV-SCT, WT-EET, KIV-EET) revealed significant positive correlations of BDNF levels between serum and thymus (coefficient *r* = 0.76), lung and spleen (*r* = 0.68), or liver and intestine (*r* = 0.64) (*p* < 0.05 for each, Fig. 4). Interestingly, significant negative correlations were observed between hippocampus and heart (*r* = −0.52), liver (*r* = −0.51), or intestine (*r* = −0.45), between frontal cortex and intestine (*r* = −0.45), and between liver and muscle (*r* = −0.42) (*p* < 0.05 for each, Fig. 4). The results indicated that the lower the levels of BDNF in the hippocampus or frontal cortex, the higher the levels of BDNF in the liver or intestine.

**Fig. 5:**
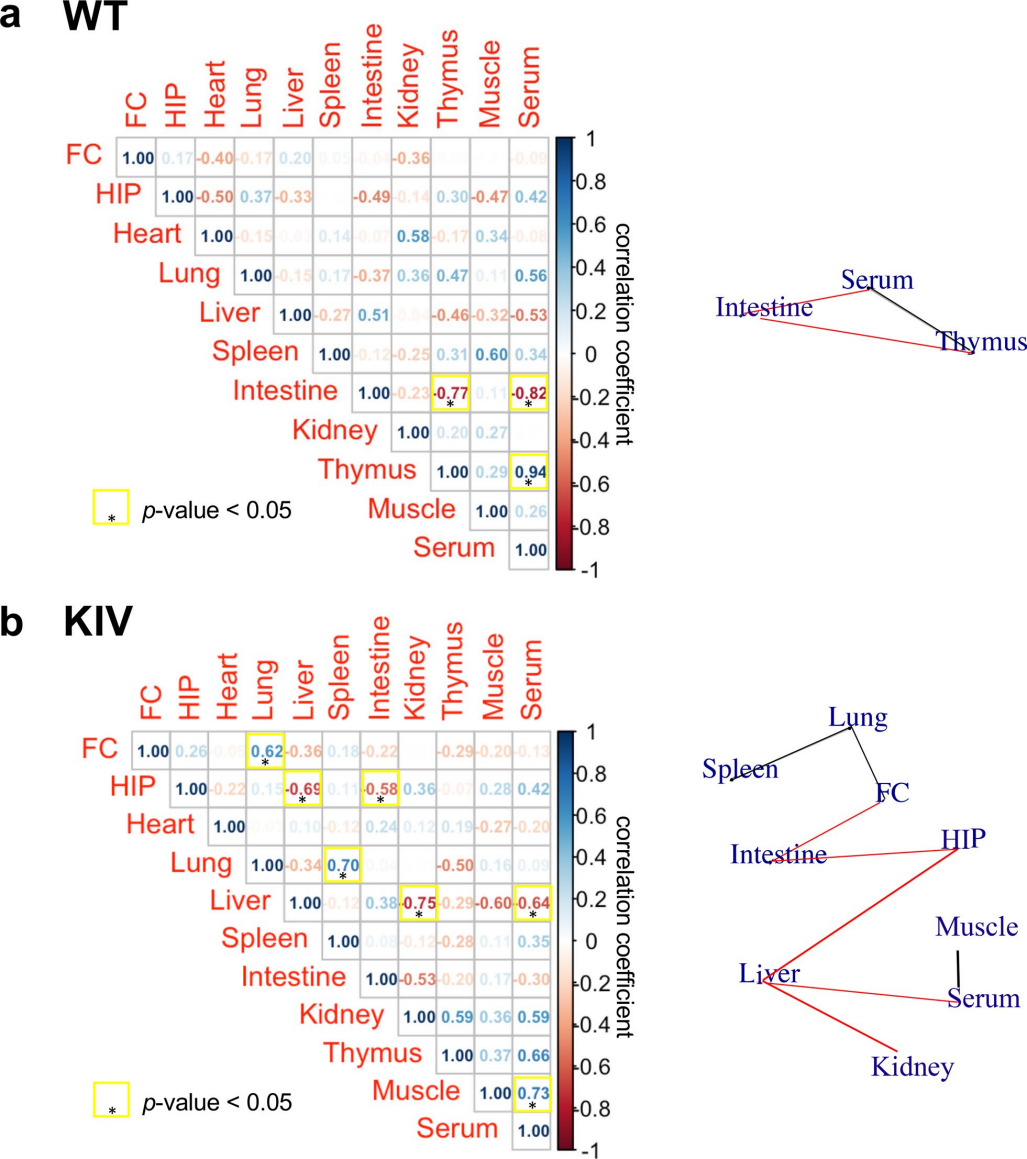
BDNF correlations across regions in (a) WT or (b) KIV mice. Pearson's correlation analyses were conducted for BDNF levels with both SCT and EET. Significant positive or negative correlations are connected by lines in black or red, respectively. N = 12 mice/genotype. *HIP* hippocampus, *FC* frontal cortex

When correlations were examined using only WT groups, results revealed a significant positive correlation between thymus and serum (*r* = 0.94) and significant negative correlations between intestine and serum (*r* = −0.82) or thymus (*r* = −0.77) (*p* < 0.05 for each, Fig. 5a, Supplementary Fig. 3a). Correlations analyses using only KIV groups revealed significant positive correlations between muscle and serum (*r* = 0.73) and between lung and spleen (*r* = 0.70) or frontal cortex (*r* = 0.62) and significant negative correlations between hippocampus and liver (*r* = −0.69) or intestine (*r* = −0.58), and between liver and serum (*r* = −0.75) or kidney (*r* = −0.75) (*p* < 0.05 for each, Fig. 5b, Supplementary Fig. 3b).

**Fig. 6:**
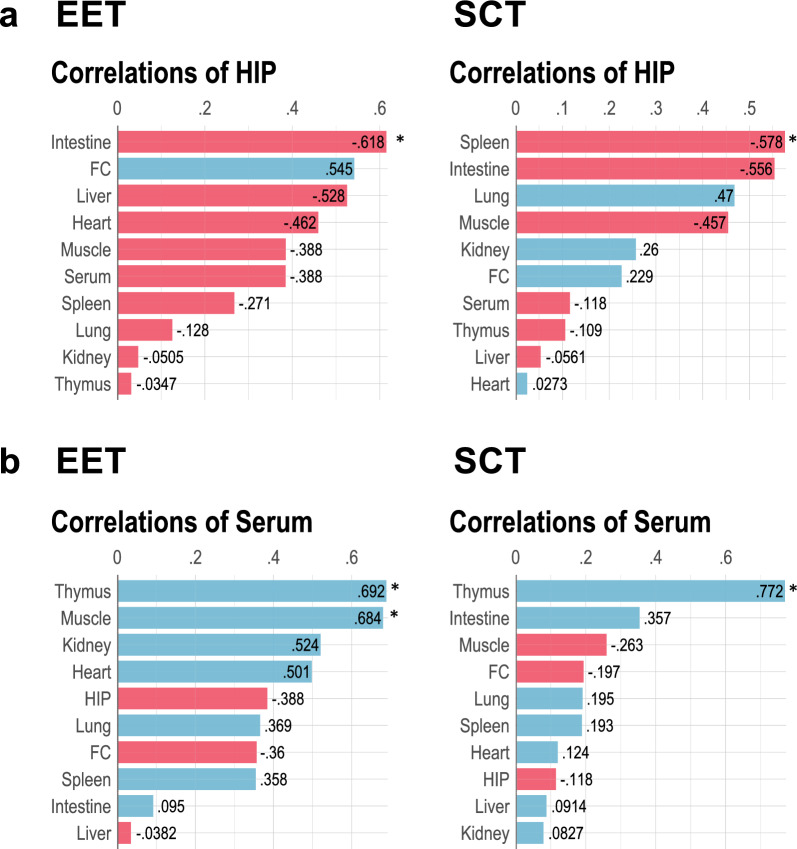
BDNF correlations in SCT and EET groups. Pearson's correlation analyses for (a) hippocampus or (b) serum were conducted for BDNF levels for EET groups or SCT groups including both WT and KIV mice. Significant positive or negative correlations are shown in blue or red, respectively. *HIP* hippocampus. N = 12 mice/treatment. *p < 0.05

We further examined whether BDNF levels in the hippocampus or serum correlate with BDNF levels in any tissues particularly in EET groups, because these BDNF levels were increased by EET (Fig. 3). Surprisingly, within EET groups, hippocampal BDNF levels were negatively correlated with BDNF levels of most of the tissues except frontal cortex, with a significance in the intestine (*r* = −0.62, *p* < 0.05, Fig. 6a, left). Hippocampal BDNF levels in SCT groups were also negatively correlated with BDNF levels in the spleen (*r* = −0.58, *p* < 0.05, Fig. 6a, right). Serum BDNF levels were positively correlated with BDNF levels in the thymus (*r* = 0.69) or muscle (*r* = 0.68) in the EET group (*p* < 0.05 for each, Fig. 6b, left). A significant serum-thymus BDNF correlation was also observed in the SCT group (*r* = 0.77, *p* < 0.05, Fig. 6b, right).

**Fig. 7:**
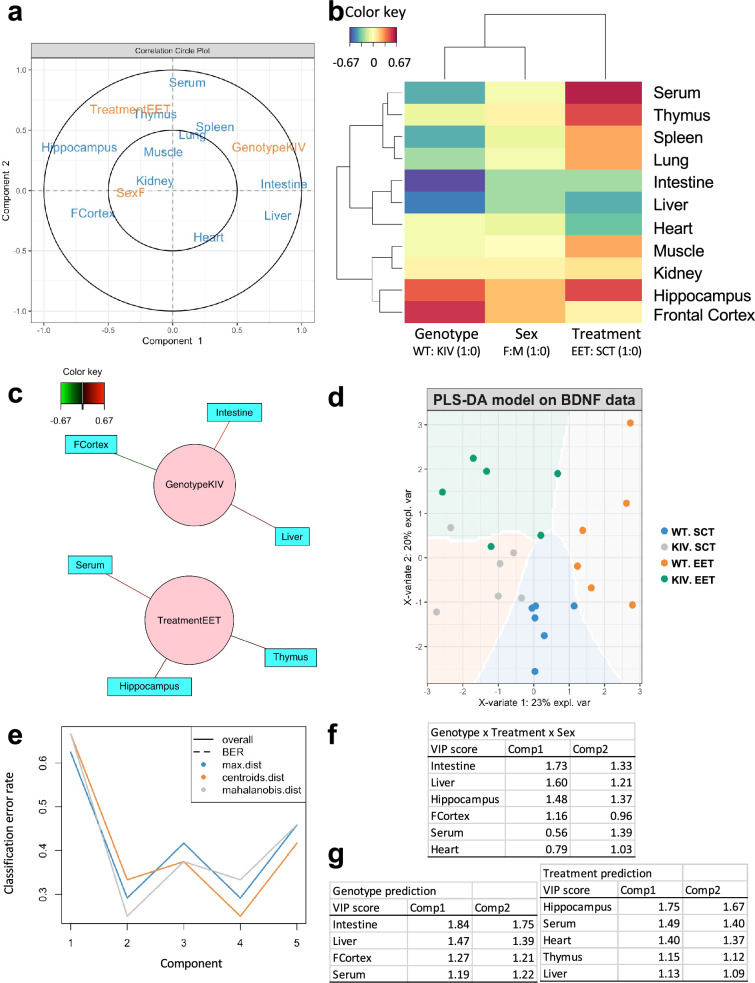
Partial Least Squares (PLS) regression model on tissue BDNF correlations to predict promoter IV defect and environments. (a) Correlation circle plot from PLS regression analyses to indicate the most important variables. Variables far from the center contribute more to the top two PLS components (largest variance). Variables closer to the center have less influence on the variation in the dataset. Variables closer together with a small angle between their vectors are positively correlated, while those with a large angle or positioned oppositely are negatively correlated. (b) Clustered image map on BDNF correlations across tissues. Similarity values and correlations between variables across two dimensions were clustered with a complete Euclidean distance method. The same colors indicate correlation in a similar direction, whereas opposing colors (red vs blue) indicate an opposite relationship. (c) Network representation showing a similarity matrix (0.45 cutoff). (d) Partial Least Squares discrimination analyses (PLS-DA) for classification model creation and validation. A prediction background (by the Mahalanobis distance metric) shows the classes that would be assigned to novel data points given their values on the first two latent components. (e) Cross-validation to find the optimal number of components. (f) Variable Importance in Projection (VIP) scores (> 1) indicate variables that significantly contribute to group separation among genotypes, treatments, and sexes; higher scores indicate more contribution. (g) VIP scores for genotype predictions and environment predictions. *WT* wild-type; *KIV* knock-in BDNF promoter IV: *SCT* standard control treatment; *EET* enriched environment treatment; *Comp* component

Finally, we used multivariable analyses to test whether the combinations of BDNF correlations among tissues may predict the genotype and treatment effects. First, the Partial Least Squares (PLS) regression analysis, which combines features of principal component analyses and multiple linear regression, was used to identify the most contributing variables and their correlational relationships. The correlation circle plot revealed that the most contributing variables (outer circle) were treatment, genotype, intestine, hippocampus, liver, and serum, while the least contributing variables were sex and kidney (Fig. 7a). Close proximity on this plot indicated positive correlations between intestine and liver, serum and thymus, or hippocampus and frontal cortex, while the opposite distant locations indicated negative correlations between hippocampus and intestine or liver (Fig. 7a). When the similarity in correlations between variables across two dimensions were calculated (Fig. 7b), clustering was observed between hippocampus and frontal cortex and between intestine and liver, which were oppositely related for depicting genotype effects. Clustering was also observed between serum and thymus, which showed similar direction with hippocampus for depicting the environmental treatment effects (Fig. 7b). The similarity matrix clarified links between genotype (KIV) factor and intestine (positive), liver (positive), or frontal cortex (negative), and links between treatment (EET) factor and hippocampus, thymus, or serum (all positive), whereas no links were detected between sex and any tissues (0.45 cutoff) (Fig. 7c).

Further, we used Partial Least Squares Discriminant Analysis (PLS-DA) to explore classification model creation and validation. The result showed clear separation for the genotypes and treatments in the mixed model (Fig. 7d), suggesting that BDNF correlations across multiple tissues may be able to predict genotype (promoter IV defect) and environment (enriched) conditions. The cross-validation analyses indicated the optimal number of the components to be 2 (Fig. 7e), while the error rate was ~ 0.3. We conducted Variable Importance to the Projection (VIP) analyses to assess which tissue BDNF variables contribute most to class separation in the prediction model. The VIP scores (> 1) identified the intestine, liver, hippocampus, frontal cortex, serum and heart as major contributing factors for the classification model in the mixed genotype x treatment x sex model (Fig. 7f). When VIP analyses were conducted separately for genotype only or treatment only, the VIP scores indicated intestine, liver, frontal cortex, and serum for genotype prediction, and hippocampus, serum, heart, thymus, and liver for treatment prediction (Fig. 7g). These results were consistent with our results of genotype effects (Fig. 2), treatment effects (Fig. 3), and canonical correlation analyses (Figs. 4, 5, 6).

## Discussion

The main findings of this study were that 1) lack of promoter IV-driven BDNF expression in KIV mice resulted in BDNF levels significantly reduced in the muscle, but significantly increased in the intestine, liver, thymus, and serum; 2) enriched environment treatment (EET) increased BDNF levels in the hippocampus of both WT and KIV mice, in the thymus of WT mice, and in the muscle and serum of KIV mice, while EET reduced BDNF levels in the heart of WT mice; 3) BDNF levels were positively correlated between serum and thymus, between intestine and liver, or between lung and spleen, and were negatively correlated between hippocampus and intestine, liver, or heart; 4) within EET group, hippocampal BDNF were negatively correlated with intestine BDNF levels, and serum BDNF levels were positively correlated with BDNF levels in the thymus and muscle, but no positive BDNF correlation was observed between hippocampus and serum; and 5) multivariate PLS-DA analyses indicated that BDNF levels in the intestine, liver, frontal cortex, and serum may contribute to genotype prediction, and those in the hippocampus, serum, heart, thymus, and liver may contribute to treatment prediction.

### Peripheral distribution of BDNF

Our results of peripheral distribution of BDNF expression, i.e., relatively higher levels in the lung, moderate levels in the heart and thymus, and kidney, and lower levels in the liver, spleen, and muscle, are largely consistent with past reports of peripheral distribution of BDNF (Aid et al. 2007; Lommatzsch et al. 1999; Esvald et al. 2023; Human Protein Atlas 2024; National Library of Medicine 2024). Its wide distribution suggests varied roles of BDNF in neurons innervating the peripheral tissues and in non-neuronal cells. Peripheral non-neuronal cells have also been reported to express BDNF, including respiratory and visceral epithelium cells (Lommatzsch et al. 1999), vascular smooth muscle (Donovan et al. 1995) and endothelial cells (Nakahashi et al. 2000), thymocytes (Lommatzsch et al. 2005; Kruse et al. 2007; Berzi et al. 2008), hepatocytes (Cassiman et al. 2001), splenocytes (Kruse et al. 2007), skeletal muscle cells (Matthews et al. 2009; Gomez-Pinilla et al. 2001), platelets (Yamamoto and Gurney 1990), and lymphocytes and monocytes (Kruse et al. 2007; Kerschensteiner et al. 1999). Compared to the well-studied roles of BDNF in neurons in the central nervous system (CNS), such as in neurogenesis, neuronal differentiation, and synaptic plasticity, peripheral roles of BDNF are yet to be established.

Our results showed that serum BDNF levels were the lowest among the tissues, but detectable (WT-SCT: 1.05 ± 0.09 pg/mg protein, 92.0 ± 6.7 pg/mL; KIV-SCT: 1.30 ± 0.08 pg/mg protein, 111.6 ± 9.2 pg/mL; WT-EET: 1.92 ± 0.48 pg/mg protein, 97.7 ± 6.9 pg/mL; KIV-EET: 1.78 ± 0.13 pg/mg protein, 145.8 ± 9.0 pg/mL). This result agreed with the previous reports that serum BDNF levels in mice are limited (Radka et al. 1996; Klein et al. 2011) but detectable (Want et al. 2023), and are approximately 100–200 times lower than serum BDNF levels in humans (10–30 ng/mL) (Karege et al. 2005; Rosenfeld et al. 1995).

## Effects of promoter IV-driven BDNF in peripheral tissues

The significant reduction of BDNF in the KIV muscle (vs. WT muscle) indicated that promoter IV significantly contributes to BDNF expression in the muscle. It is possible that promoter IV, a calcium-sensitive activity-dependent promoter (Tao et al. 1998), regulates the activity-induced plasticity at the neuro-muscular junctions and muscle development (Lohof et al. 1993; Wang et al. 1995).

Unexpectedly, KIV mice, which lack promoter IV-driven BDNF expression, showed significantly increased levels of BDNF in the intestine, liver, thymus, and serum, compared to WT mice. The results suggest compensatory upregulation of BDNF driven by other promoters in these peripheral tissues. Indeed, BDNF expression in these tissues is predominantly regulated by promoters other than promoter IV; for example, by promoters I and III in the thymus, by promoters V and VIII in the intestine, and by promoters V, VI, and VIII in the liver (Aid et al. 2007; Esvald et al. 2023; Kruse et al. 2007; Liu et al. 2006). Serum BDNF is partly derived from platelets, and in the platelet progenitors, megakaryocytes, BDNF mRNA expression is predominantly driven by promoter IXa in mice (Chacon-Fernandez et al. 2016). The mechanisms of BDNF upregulation in these peripheral tissues and serum of KIV mice remain unknown, but such upregulation may be to compensate for the lower BDNF levels in the central nervous system responding to neuronal activity (Sakata et al. 2009; Sakata et al. 2013a). The peripheral BDNF upregulation may also be caused by decreased inhibitory functions in the central nervous system due to promoter IV defect (Sakata et al. 2009; Hong et al. 2008).

## Negative BDNF correlations between hippocampus and peripheral tissues

Interestingly, BDNF levels showed significant negative correlations between hippocampus and the digestive tissues, i.e., intestine and liver, particularly in KIV mice, while there was a significant positive correlation between intestine and liver (Fig. 5b, Supplementary Fig. 3b). The results suggest a brain-gut-liver regulation that controls BDNF levels. In the intestine, BDNF is expressed at the epithelium, smooth muscles, and enteric neuronal ganglia (Lommatzsch et al. 1999; Boesmans et al. 2008; Hoehner et al. 1996), regulating vagal neuron innervation and gut barrier integrity (Murphy and Fox 2010; Biddinger and Fox 2014; Zhao et al. 2018). Functionally, BDNF enhances cholinergic smooth muscle contraction (Al-Qudah et al. 2014) and stimulates gut motility (Coulie et al. 2000). BDNF deletion specifically in intestinal smooth muscle causes decreased food intake (Biddinger and Fox 2014). Our results of increased BDNF expression in the KIV intestine suggest increased intestinal movement and food intake in KIV mice. Chronic excess of BDNF in the KIV intestine may lead to irritable bowel syndrome (IBS), a chronic disorder characterized by recurrent abdominal pain, visceral hyperalgesia, and abnormal bowel movements (Enck et al. 2016), as indicated by previous research reporting increased BDNF expression in IBS patients and its positive correlation with abdominal pain and disease severity (Yu et al. 2012; Zhang et al. 2019). IBS often co-occurs with depression and anxiety (Fond et al. 2014), psychiatric conditions associated with decreased BDNF expression in the brain (Dwivedi et al. 2003). Similarly, our result of increased liver BDNF levels in KIV mice is also consistent with past research suggesting that patients with psychiatric disorders typically show lower BDNF levels in the brain but higher levels in liver compared to normal controls (Yang et al. 2017). In the liver, BDNF activates catabolic pathways in hepatocytes, such as fatty acid oxidation, and inhibition of gluconeogenesis (Genzer et al. 2017), or protects hepatocytes under endoplasmic reticulum (ER) stress (Cirrik et al. 2019) (see review (Iu and Chan 2022)). Inactivated promoter IV and BDNF reduction are observed in the brain of psychiatric human patients (Keller et al. 2010; Wong et al. 2010) and chronically stressed rats (Tsankova et al. 2006; Roth et al. 2009), while pIV-BDNF ablation in KIV mice causes psychiatric-related behaviors (Sakata et al. 2009, 2010, 2013a). How promoter IV inactivation increases the peripheral BDNF levels through other promoters and how this affects peripheral functions remain to be elucidated in future studies.

**Why more changes in BDNF levels in peripheral organs than in CNS of KIV mice? **Promoter IV is a neuronal activity-dependent promoter (Metsis et al. 1993; Tao et al. 1998; Shieh et al. 1998). Its inactivation largely abolishes activity-induced expression of BDNF protein but does not affect constitutive expression in neurons (e.g., under activity inhibition by tetrodotoxin, a sodium channel blocker) (Sakata et al. 2009, 2013a). Lack of significant changes in BDNF levels in the CNS of KIV mice compared to WT mice may be explained by the circadian rhythm of promoter IV activity (Supplementary Fig. 4a), which is lowest during the late light phase (12:00–18:00) in the hippocampus of rodents (Berchtold et al. 1999). The tissues were collected before the dark phase started (~ 16:30) when nocturnal mice were sleeping (inactive) because we wanted to examine the basal levels of BDNF affected by chronic EET (3 weeks) yet relatively unaffected by acute activity of individuals (e.g., running). In other studies when the tissues were collected in the dark phase closer to the mouse active phase (in the morning or before 15:00), KIV mice showed reduced BDNF protein levels in the hippocampus compared to WT mice (Jha et al. 2011) (Sakata unpublished).

Several possible mechanisms may explain why the peripheral BDNF changes were more detected in KIV mice (Supplementary Fig. 4b). First, circadian activity may have different effects in peripheral tissues. When the brain is asleep (inactive), some peripheral organs become active (e.g., immune organs, metabolism organs, and activity-dependent muscle production). While the peripheral circadian controls of BDNF promoter activity remain largely unknown, it is possible that BDNF promoters in the peripheral organs may be more active and persist during the animal's inactive state. Another possibility is different BDNF protein kinetics across different tissues. BDNF functioning like a neurotransmitter/modulator in the CNS may be rapidly degraded, whereas in peripheral tissues, BDNF may be slowly released and degraded. For example, activity (Ca^2+^)-induced promoter IV-driven BDNF expression in muscle may persist in animal's inactive phase for muscle growth and repair, which can be detected as a reduction of BDNF protein levels in KIV muscle compared to WT muscle (Supplementary Fig. 4b, left). Furthermore, BDNF release and uptake may occur across peripheral cells. High levels of circulating BDNF in the blood may be taken up by peripheral cells (e.g., platelets, endothelial cells and hepatocytes), which may be detected as higher levels of peripheral BDNF in KIV mice. Additionally, peripheral BDNF transcription may become active by reduced GABAergic inhibitory function in the KIV CNS (Sakata et al. 2009). These possibilities can be elucidated in the future.

## EET effects

**Hippocampus BDNF:** EET increased hippocampal BDNF levels in both WT and KIV mice, reproducing our previous results and indicating neurogenic effect of EET in the hippocampus (Jha et al. 2011). Our result also agrees with others’ reports that exercise, a critical component of EET, increases BDNF levels in the hippocampus (Neeper et al. 1995; Kobilo et al. 2011). Of note, EET produced a non-significant increase in BDNF levels in males, possibly due to the small number of animals (N = 6 per group, Supplementary Fig. 2a), since EET significantly increased hippocampal BDNF levels in males when N = 12 per group were used (Jha et al. 2011). Also, the EET effects on BDNF increases were larger in females than in males in both WT and KIV mice (Supplementary Fig. 2b). The exact causes of these sex differences remain unknown but may be due to different social behaviors, such as competition and fighting in males, and estrogen exposure in females during development; estrogen increases BDNF expression (Sohrabji et al. 1995).

**Muscle BDNF:** EET significantly increased BDNF levels in the muscle of KIV mice and normalized its reduction caused by promoter IV defect (WT-EET vs KIV-EET *p* > 0.05, Supplementary Fig. 5). This result is consistent with previous studies that physical exercise increases BDNF levels in skeletal muscle (Matthews et al. 2009; Gomez-Pinilla et al. 2001; Cuppini et al. 2007; Renteria et al. 2022). In WT muscles, EET prompted a non-significant increase in BDNF protein levels (Fig. 3, Supplementary Fig. 5). Statistically significant increases may be more easily detected when basal levels of BDNF are low, like in KIV mice, while individual differences in muscle activity may account for the large data variability in muscle. Muscle BDNF expression is driven by promoter IV (Cuppini et al. 2007; Mousavi and Jasmin 2006; Hurtado et al. 2017) and other multiple promoters, such as V, VI, VII, VIII, and IXa (Pruunsild et al. 2007; Aid et al. 2007; Esvald et al. 2023; Liu et al. 2006). Our results suggest that EET can normalize the BDNF reduction caused by defective promoter IV via other promoters, similar to what we observed in the hippocampus (Jha et al. 2011). Which promoter is responsible for these EET effects remains to be elucidated. Because we used tissue homogenates, it is also unknown whether BDNF increases in the muscle cells or motor neurons. BDNF is expressed in both muscle cells (myocytes and satellite cells) and innervating motor neurons (Funakoshi et al. 1993), and is increased by neuronal activity and muscle contraction (Matthews et al. 2009; Hurtado et al. 2017). Increased muscle BDNF levels by EET can play several roles: increasing neuro-muscular junction for motor controls (Lohof et al. 1993; Wang et al. 1995), fat oxidation in skeletal muscle (Matthews et al. 2009), fasting-induced metabolic and glucose controls (Fulgenzi et al. 2015), and recovery from exercise-induced muscle damage or motor neuron regeneration (Yan et al. 1992; Koliatsos et al. 1993).

**Serum BDNF and its correlation with muscle BDNF:** Interestingly, EET significantly increased BDNF levels in serum of KIV mice (Fig. 3), and the serum BDNF levels positively correlated with muscle BDNF levels in KIV mice (Fig. 5b, Supplementary Fig. 3b) and under EET (Fig. 6b**, left**). Many studies have demonstrated that physical exercise increases blood BDNF levels (Szuhany et al. 2015; Assis and Almondes 2017), but there have been conflicting reports regarding whether this exercise-induced increase is from muscle. Matthews et al. (2009) reported that muscle-derived BDNF appeared not to be released into the blood circulation because an increase in BDNF levels in serum (2 h) after exercise preceded that in the skeletal muscle (24 h) after exercise. However, a more recent study by Fulgenzi et al. (2020) showed that skeletal muscle secreted biologically active BDNF. Our results of the correlation between muscle and serum BDNF levels suggest that EET-induced BDNF expression in muscle may be contributing to the increased serum BDNF levels. It is possible that this correlation gradually forms after chronic EET and exercise (i.e., 3 weeks). It should be noted that the correlation was observed only in KIV mice, but not in WT mice. This suggests that the muscle-serum BDNF correlation may become evident only when basal levels of muscle BDNF are reduced by promoter IV defects, such as under low physical activity, stress, or disease conditions.

The functional significance of increased serum BDNF levels remains obscure, but an interesting study by Schmidt and Duman (2010) has shown that peripheral administration of BDNF (s.c., 2 weeks) increased hippocampal neurogenesis and anxiolytic and antidepressant behavioral effects in mice. These effects could result from direct actions of BDNF that is transported into the brain via blood circulation (although endogenous BDNF unlikely easily passes the blood–brain barrier (Wu and Pardridge 1999)) or result from indirect effects of BDNF on peripheral tissues that activate the brain (e.g., via peripheral neurons or other mediating factors). Regardless of the mechanism, BDNF in the blood may be capable of producing central actions. Our results of increased serum BDNF levels in KIV mice (vs. WT mice) and its further increase by EET suggest that the serum BDNF upregulation via peripheral-dominant BDNF promoters may be to compensate for the dysfunctions caused by pIV-BDNF deficiency in the brain, such as decreased hippocampal neurogenesis and LTP (Sakata et al. 2009, 2013a; Jha et al. 2011). This is further exemplified by demonstration of a negative correlation between serum BDNF levels and hippocampal and frontal cortex BDNF levels (Fig. 6b).

**Thymus BDNF and its correlation with serum BDNF:** Interestingly, EET significantly increased thymus BDNF levels in WT mice (Fig. 3), and the highest positive correlation was observed between thymus and serum (Fig. 4, 5a, 6b) particularly in WT mice (*r* = 0.94, Supplementary Fig. 3a). EET also increased BDNF levels in KIV thymus and WT serum, but the effects did not reach significance likely due to data variation (Fig. 3). KIV mice showed a trend of positive correlation between thymus and serum (*r* = 0.66, *p* = 0.052, Supplementary Fig. 3b). These results suggest that thymus BDNF levels and circulating blood BDNF levels are coordinated. In thymus, BDNF plays a role in thymocytes survival (Garcia-Suarez et al. 2002) and their maturation to thymus-dependent lymphocytes (T cells) (Linker et al. 2015; Sharma et al. 2021). Circulatory blood BDNF also regulates lymphocytes, reducing their apoptosis and enhancing their proliferation (Wang and Tian 2021). EET has been reported to regulate thymocyte development and alleviates autoimmune responses in encephalomyelitis mouse model, while hypothalamic BDNF is reported to mediate the EET’s thymic effects (Xiao et al. 2019). The exact source of serum BDNF remains elusive, but blood cells are suggested to be partly involved because activated platelets and white blood cells (T cells, B cells, and macrophages) release BDNF (Yamamoto and Gurney 1990; Kerschensteiner et al. 1999; Fujimura et al. 2002). Our results of EET upregulating thymus BDNF levels and the positive thymus-serum BDNF correlation suggest that thymus may be a part of BDNF upregulation in the serum, or vice versa. EET-induced BDNF may control thymocytes and T cells to enhance immunity while suppressing autoimmune reactions, although the specific functional mechanisms remain to be elucidated.

**EET modulates correlation between hippocampal and peripheral BDNF:** Contrary to our hypothesis, hippocampal BDNF levels, which were upregulated by EET, were inversely correlated with BDNF levels of most peripheral tissues, and with a significance with intestine BDNF levels under EET (Fig. 6a**, left**). The result suggests that EET-induced increases in hippocampal BDNF (Fig. 3) may suppress BDNF levels in the intestine. It should also be noted that intestine BDNF levels were negatively correlated with thymus or serum BDNF levels in WT mice (Fig. 5a, Supplementary Fig. 3a), while EET increased thymus BDNF levels in WT mice (Fig. 2). The results suggest that a regulatory system exists to suppress intestine BDNF expression when BDNF levels are increased in the hippocampus, thymus, or serum. As a note, no significant BDNF correlation was observed between the hippocampus and thymus or serum (Fig. 4, 5, 6), suggesting that BDNF upregulation by EET was independent between hippocampus and thymus or serum.

**EET reduced heart BDNF:** EET unexpectedly decreased BDNF levels in the heart of WT mice. Our results contrasted previous research showing that exercise, an EET component, increased BDNF levels in the heart in rats (Wang et al. 2019) and mice (Yang et al. 2023). This difference may be due to the differences in the treatment, i.e., EET versus exercise, voluntary exercise on a running wheel versus forced exercise on a treadmill, and time of tissue collection after the treatment. BDNF plays a role in cardiomyocytes for homeostatic heart function (Pius-Sadowska and Machalinski 2017). Intracerebroventricular infusion of BDNF reduces heart rate by cardioinhibitory parasympathetic activity (Wan et al. 2014). Possibly, EET increased BDNF levels in the brain (hippocampus) or blood (serum), which may have caused a parasympathetic cardio-inhibition that reduces heart rate and contractility, and a subsequent reduction in the heart BDNF.

**BDNF correlations in other tissues:** Our results showed several other BDNF correlations, particularly in KIV mice, including positive BDNF correlations between lung and spleen or frontal cortex, negative correlations between liver and kidney or serum, or between hippocampus and spleen, of which mechanisms remain to be studied.

**Predicting promoter IV defects and EET effects by BDNF tissue correlation:** The results from multivariate analyses (Fig. 7) confirmed the results from canonical correlation analyses (Fig. 3–6) and suggested that a multivariate model on BDNF levels may predict the genotype x treatment effects. Specifically, our results identified intestine, liver, frontal cortex and serum for predicting promoter IV defects, and hippocampus, serum heart, thymus, and liver for predicting treatment effects. The current model has a high error rate (0.3), indicating more samples are needed to verify the model. Future similar studies can verify whether the combinations of BDNF correlations among several tissues, rather than from one tissue (e.g., only in serum), may better predict BDNF promoter defect and treatment effects. Understanding cross-tissue BDNF correlations may also help clarify the complex inter-organ BDNF regulation and its roles.

## Limitations and future directions

This study has several limitations. First, species differences need to be considered. For example, mouse serum BDNF levels are much lower than those in humans, and mice platelets are not the source of BDNF found in blood (Want et al. 2023), while in other species, like rats and humans, platelets are thought to be one major source of serum BDNF (Rosenfeld et al. 1995). The peripheral effects of promoter IV and EET may differ by species and the BDNF correlations observed in mice may not apply to those in humans.

Second, KIV mice are created by genetic manipulation, and thus observed changes in BDNF levels may not reflect physiological changes. In KIV mice, the GFP/PGK-promoter-Neo cassette was inserted to block promoter IV-driven BDNF protein expression (Sakata et al. 2009). This insertion may cause artifacts in gene regulation due to changes in the 3-dimensional DNA structure and enhancer binding. However, the inserted PGK promoter unlikely contributed to the increases in peripheral BDNF levels because: 1) PGK promoter was inserted into *Bdnf* exon IV in the direction to drive antisense transcription; 2) PGK promoter is primarily unidirectional driving transcription of downstream DNA (5' to 3') and not upstream (3' to 5') (Johnson and Friedmann 1990) and any 3'-to-5' transcription would terminate within a few (~ 2.5 kb) kilobases (Preker et al. 2008), not reaching BDNF-protein-coding exon IX located at its 30 kb upstream; and 3) in the second generation of KIV mice (e4, (Maynard et al. 2016)) where the PGK promoter-Neo cassette was removed, increased levels of serum BDNF protein were also observed (Supplementary Fig. 6a). A result of muscle BDNF reduction was also reproduced in e4 mice (Supplementary Fig. 6b). Nevertheless, any artifacts by the inserted cassette (including GFP), such as altered 3-dimensional DNA structure affecting BDNF gene regulation, can be addressed by studies using other BDNF mutant models (e.g., promoter IV mutation model (Hong et al. 2008)) and methods (e.g., viral BDNF knock-down). While each approach has its limitation (e.g., limited blockade of activity-induced BDNF in the CNS), future comprehensive studies together with related physiological models (e.g., chronic stress models) may address this limitation.

Third, our data number for the correlation analyses is low. We used only half of the data collected because we noticed a ‘plate factor,’ i.e., different ELISA plates showed variations in data even from the same samples, which could cause an artificial result (i.e., showing always positive correlations). Thus, to examine the correlations between different tissues, we only used data from plates where all 11 different tissues from the same mouse were measured on the same plate. Such technical issues can be addressed in the future (e.g., technical advances to do ELISA in 386 wells). Analyses with increased numbers of samples may provide better accuracy and detection for BDNF correlations among different tissues and help to create a model that predicts the promoter IV defect and environmental effects.

Finally, our results of BDNF correlations predict BDNF regulation, such as in the brain-gut-liver axis and the thymus-serum or serum-muscle axis, but the causal relationship remains unknown. The mechanisms leading to the peripheral BDNF changes and its roles need to be elucidated in future studies, which may help understand comorbid peripheral phenotypes in psychiatric disorders with CNS BDNF deficiency.

## Conclusion

This is the first study that comprehensively examined the causal relationships of how genetic (promoter IV defect) and environmental (EET) manipulations affected BDNF levels across brain and peripheral tissues. Our results supported our hypothesis that promoter IV defect and EET affected peripheral BDNF levels, but the correlations between brain and peripheral BDNF levels were rather complex: i.e., there was no positive hippocampus-peripheral correlation, but there was a negative hippocampus-intestine correlation under EET. There were also negative intestine-thymus or intestine-serum correlations, while there were positive thymus-serum, serum-muscle, intestine-liver, and spleen-lung correlations. The deficiency of promoter IV-driven BDNF caused BDNF decreases in muscle, but caused BDNF increases in the liver, intestine, thymus, and serum through possible compensatory mechanisms, which may affect peripheral functions (e.g., intestinal movement and pain). EET upregulating BDNF expression in muscle, serum, thymus, and hippocampus, suggests its beneficial effects in muscle motor control and adaptive immunity, as well as in hippocampal neurogenesis and neuronal plasticity. BDNF expression in the periphery likely plays various roles, not only in the normal function of organs, but also in relation to central BDNF levels. Further development of multivariate BDNF models across tissues may help better predict the BDNF genetic and treatment effects and elucidate the inter-organ BDNF regulatory pathways. This may help us understand the complex peripheral connection to CNS disorders such as depression. We hope that by uncovering these pathways, we will be able to use BDNF as biomarker more effectively for the detection of CNS disorders and the assessment of treatment efficacy and disease remission.

## Supplementary Information

Supplementary Fig. 1-6.

Supplementary Table 1.

## Data Availability

The data are available in the article and its supplementary material. Materials are available from the corresponding author upon reasonable request.

## References

[CR1] Aarons T, et al. Dysregulation of BDNF in prefrontal cortex in Alzheimer’s disease. J Alzheimers Dis. 2019;69:1089–97. 31127785 10.3233/JAD-190049

[CR2] Aid T, Kazantseva A, Piirsoo M, Palm K, Timmusk T. Mouse and rat BDNF gene structure and expression revisited. J Neurosci Res. 2007;85:525–35. 17149751 10.1002/jnr.21139 PMC1878509

[CR3] Al-Qudah M, et al. Brain-derived neurotrophic factor enhances cholinergic contraction of longitudinal muscle of rabbit intestine via activation of phospholipase C. Am J Physiol Gastrointest Liver Physiol. 2014;306:G328-337. 24356881 10.1152/ajpgi.00203.2013 PMC3920121

[CR5] Berchtold NC, Oliff HS, Isackson P, Cotman CW. Hippocampal BDNF mRNA shows a diurnal regulation, primarily in the exon III transcript. Brain Res Mol Brain Res. 1999;71:11–22. 10407182 10.1016/s0169-328x(99)00137-0

[CR6] Berzi A, et al. BDNF and its receptors in human myasthenic thymus: implications for cell fate in thymic pathology. J Neuroimmunol. 2008;197:128–39. 18555538 10.1016/j.jneuroim.2008.04.019

[CR7] Biddinger JE, Fox EA. Reduced intestinal brain-derived neurotrophic factor increases vagal sensory innervation of the intestine and enhances satiation. J Neurosci. 2014;34:10379–93. 25080597 10.1523/JNEUROSCI.1042-14.2014 PMC4115142

[CR8] Boesmans W, Gomes P, Janssens J, Tack J, Vanden BP. Brain-derived neurotrophic factor amplifies neurotransmitter responses and promotes synaptic communication in the enteric nervous system. Gut. 2008;57:314–22. 17965066 10.1136/gut.2007.131839

[CR9] Cassiman D, Denef C, Desmet VJ, Roskams T. Human and rat hepatic stellate cells express neurotrophins and neurotrophin receptors. Hepatology. 2001;33:148–58. 11124831 10.1053/jhep.2001.20793

[CR10] Chacon-Fernandez P, et al. Brain-derived neurotrophic factor in megakaryocytes. J Biol Chem. 2016;291:9872–81. 27006395 10.1074/jbc.M116.720029 PMC4858990

[CR11] Cirrik S, Hacioglu G, Abidin I, Aydin-Abidin S, Noyan T. Endoplasmic reticulum stress in the livers of BDNF heterozygous knockout mice. Arch Physiol Biochem. 2019;125:378–86. 30039987 10.1080/13813455.2018.1489850

[CR12] Connor B, et al. Brain-derived neurotrophic factor is reduced in Alzheimer’s disease. Brain Res Mol Brain Res. 1997;49:71–81. 9387865 10.1016/s0169-328x(97)00125-3

[CR13] Coulie B, et al. Recombinant human neurotrophic factors accelerate colonic transit and relieve constipation in humans. Gastroenterology. 2000;119:41–50. 10889153 10.1053/gast.2000.8553

[CR14] Cuppini R, et al. Bdnf expression in rat skeletal muscle after acute or repeated exercise. Arch Ital Biol. 2007;145:99–110. 17639782

[CR15] de Assis GG, de Almondes KM. Exercise-dependent BDNF as a modulatory factor for the executive processing of individuals in course of cognitive decline. Syst Rev Front Psychol. 2017;8:584. 10.3389/fpsyg.2017.00584 PMC5395613 28469588

[CR16] Dong BE, Xue Y, Sakata K. The effect of enriched environment across ages: a study of anhedonia and BDNF gene induction. Genes Brain Behav. 2018;17:e12485. 29717802 10.1111/gbb.12485 PMC6214784

[CR17] Dong BE, Chen H, Sakata K. BDNF deficiency and enriched environment treatment affect neurotransmitter gene expression differently across ages. J Neurochem. 2020;154:41–55. 32222968 10.1111/jnc.15017 PMC7319906

[CR18] Donovan MJ, et al. Neurotrophin and neurotrophin receptors in vascular smooth muscle cells. Regulation of expression in response to injury. Am J Pathol. 1995;147:309–24. 7639328 PMC1869811

[CR19] Dwivedi Y, et al. Altered gene expression of brain-derived neurotrophic factor and receptor tyrosine kinase B in postmortem brain of suicide subjects. Arch Gen Psychiatry. 2003;60:804–15. 12912764 10.1001/archpsyc.60.8.804

[CR20] Enck P, et al. Irritable bowel syndrome. Nat Rev Dis Primers. 2016;2:16014. 27159638 10.1038/nrdp.2016.14 PMC5001845

[CR21] Esvald EE, et al. Revisiting the expression of BDNF and its receptors in mammalian development. Front Mol Neurosci. 2023;16:1182499. 37426074 10.3389/fnmol.2023.1182499 PMC10325033

[CR22] Fernandes BS, et al. Peripheral brain-derived neurotrophic factor in schizophrenia and the role of antipsychotics: meta-analysis and implications. Mol Psychiatry. 2015;20:1108–19. 25266124 10.1038/mp.2014.117

[CR23] Fond G, et al. Anxiety and depression comorbidities in irritable bowel syndrome (IBS): a systematic review and meta-analysis. Eur Arch Psychiatry Clin Neurosci. 2014;264:651–60. 24705634 10.1007/s00406-014-0502-z

[CR24] Fujimura H, et al. Brain-derived neurotrophic factor is stored in human platelets and released by agonist stimulation. Thromb Haemost. 2002;87:728–34. 12008958

[CR25] Fulgenzi G, et al. BDNF modulates heart contraction force and long-term homeostasis through truncated TrkB.T1 receptor activation. J Cell Biol. 2015;210:1003–12. 26347138 10.1083/jcb.201502100 PMC4576863

[CR26] Fulgenzi G, et al. Novel metabolic role for BDNF in pancreatic beta-cell insulin secretion. Nat Commun. 2020;11:1950. 32327658 10.1038/s41467-020-15833-5 PMC7181656

[CR27] Funakoshi H, et al. Differential expression of mRNAs for neurotrophins and their receptors after axotomy of the sciatic nerve. J Cell Biol. 1993;123:455–65. 8408225 10.1083/jcb.123.2.455 PMC2119843

[CR28] Garcia-Suarez O, et al. Massive lymphocyte apoptosis in the thymus of functionally deficient TrkB mice. J Neuroimmunol. 2002;129:25–34. 12161017 10.1016/s0165-5728(02)00166-2

[CR29] Genzer Y, Chapnik N, Froy O. Effect of brain-derived neurotrophic factor (BDNF) on hepatocyte metabolism. Int J Biochem Cell Biol. 2017;88:69–74. 28483667 10.1016/j.biocel.2017.05.008

[CR30] Gomez-Pinilla F, Ying Z, Opazo P, Roy RR, Edgerton VR. Differential regulation by exercise of BDNF and NT-3 in rat spinal cord and skeletal muscle. Eur J Neurosci. 2001;13:1078–84. 11285004 10.1046/j.0953-816x.2001.01484.x

[CR31] Hashimoto T, et al. Relationship of brain-derived neurotrophic factor and its receptor TrkB to altered inhibitory prefrontal circuitry in schizophrenia. J Neurosci. 2005;25:372–83. 15647480 10.1523/JNEUROSCI.4035-04.2005 PMC6725470

[CR32] Hing B, et al. A polymorphism associated with depressive disorders differentially regulates brain derived neurotrophic factor promoter IV activity. Biol Psychiatry. 2012;71:618–26. 22265241 10.1016/j.biopsych.2011.11.030 PMC3712170

[CR33] Hoehner JC, Wester T, Pahlman S, Olsen L. Localization of neurotrophins and their high-affinity receptors during human enteric nervous system development. Gastroenterology. 1996;110:756–67. 8608885 10.1053/gast.1996.v110.pm8608885

[CR34] Hong EJ, McCord AE, Greenberg ME. A biological function for the neuronal activity-dependent component of Bdnf transcription in the development of cortical inhibition. Neuron. 2008;60:610–24. 19038219 10.1016/j.neuron.2008.09.024 PMC2873221

[CR4] Human Protein Atlas. BDNF tissue RNA expression. 2024. https://www.proteinatlas.org/ENSG00000176697-BDNF/tissue#rna_expression.

[CR35] Hurtado E, et al. Muscle contraction regulates BDNF/TrkB Signaling to modulate synaptic function through presynaptic cPKCalpha and cPKCbetaI. Front Mol Neurosci. 2017;10:147. 28572757 10.3389/fnmol.2017.00147 PMC5436293

[CR36] Iu ECY, Chan CB. Is brain-derived neurotrophic factor a metabolic hormone in peripheral tissues? Biology (Basel). 2022. 10.3390/biology11071063. 36101441 10.3390/biology11071063 PMC9312804

[CR37] Jha S, Dong B, Sakata K. Enriched environment treatment reverses depression-like behavior and restores reduced hippocampal neurogenesis and protein levels of brain-derived neurotrophic factor in mice lacking its expression through promoter IV. Transl Psychiatry. 2011;1: e40. 22832656 10.1038/tp.2011.33 PMC3309483

[CR38] Jha S, et al. Antidepressive and BDNF effects of enriched environment treatment across ages in mice lacking BDNF expression through promoter IV. Transl Psychiatry. 2016;6: e896. 27648918 10.1038/tp.2016.160 PMC5048201

[CR39] Johnson P, Friedmann T. Limited bidirectional activity of two housekeeping gene promoters: human HPRT and PGK. Gene. 1990;88:207–13. 2347494 10.1016/0378-1119(90)90033-n

[CR40] Karege F, et al. Decreased serum brain-derived neurotrophic factor levels in major depressed patients. Psychiatry Res. 2002;109:143–8. 11927139 10.1016/s0165-1781(02)00005-7

[CR41] Karege F, et al. Low brain-derived neurotrophic factor (BDNF) levels in serum of depressed patients probably results from lowered platelet BDNF release unrelated to platelet reactivity. Biol Psychiatry. 2005;57:1068–72. 15860348 10.1016/j.biopsych.2005.01.008

[CR42] Keller S, et al. Increased BDNF promoter methylation in the Wernicke area of suicide subjects. Arch Gen Psychiatry. 2010;67:258–67. 20194826 10.1001/archgenpsychiatry.2010.9

[CR43] Kerschensteiner M, et al. Activated human T cells, B cells, and monocytes produce brain-derived neurotrophic factor in vitro and in inflammatory brain lesions: a neuroprotective role of inflammation? J Exp Med. 1999;189:865–70. 10049950 10.1084/jem.189.5.865 PMC2192942

[CR44] Klein AB, et al. Blood BDNF concentrations reflect brain-tissue BDNF levels across species. Int J Neuropsychopharmacol. 2011;14:347–53. 20604989 10.1017/S1461145710000738

[CR45] Kobilo T, et al. Running is the neurogenic and neurotrophic stimulus in environmental enrichment. Learn Mem. 2011;18:605–9. 21878528 10.1101/lm.2283011 PMC3166785

[CR46] Koliatsos VE, Clatterbuck RE, Winslow JW, Cayouette MH, Price DL. Evidence that brain-derived neurotrophic factor is a trophic factor for motor neurons in vivo. Neuron. 1993;10:359–67. 8080464 10.1016/0896-6273(93)90326-m

[CR47] Kruse N, Cetin S, Chan A, Gold R, Luhder F. Differential expression of BDNF mRNA splice variants in mouse brain and immune cells. J Neuroimmunol. 2007;182:13–21. 17046071 10.1016/j.jneuroim.2006.09.001

[CR48] Linker RA, et al. Thymocyte-derived BDNF influences T-cell maturation at the DN3/DN4 transition stage. Eur J Immunol. 2015;45:1326–38. 25627579 10.1002/eji.201444985

[CR49] Liu QR, et al. Rodent BDNF genes, novel promoters, novel splice variants, and regulation by cocaine. Brain Res. 2006;1067:1–12. 16376315 10.1016/j.brainres.2005.10.004

[CR50] Lohof AM, Ip NY, Poo MM. Potentiation of developing neuromuscular synapses by the neurotrophins NT-3 and BDNF. Nature. 1993;363:350–3. 8497318 10.1038/363350a0

[CR51] Lommatzsch M, et al. Abundant production of brain-derived neurotrophic factor by adult visceral epithelia. Implications for paracrine and target-derived Neurotrophic functions. Am J Pathol. 1999;155:1183–93. 10514401 10.1016/S0002-9440(10)65221-2 PMC1867012

[CR52] Lommatzsch M, et al. Neurotrophins in murine viscera: a dynamic pattern from birth to adulthood. Int J Dev Neurosci. 2005;23:495–500. 15978771 10.1016/j.ijdevneu.2005.05.009

[CR53] Lu B. BDNF and activity-dependent synaptic modulation. Learn Mem. 2003;10:86–98. 12663747 10.1101/lm.54603 PMC5479144

[CR54] Matthews VB, et al. Brain-derived neurotrophic factor is produced by skeletal muscle cells in response to contraction and enhances fat oxidation via activation of AMP-activated protein kinase. Diabetologia. 2009;52:1409–18. 19387610 10.1007/s00125-009-1364-1

[CR55] Maynard KR, et al. Functional role of BDNF production from unique promoters in aggression and serotonin signaling. Neuropsychopharmacology. 2016;41:1943–55. 26585288 10.1038/npp.2015.349 PMC4908631

[CR57] Metsis M, Timmusk T, Arenas E, Persson H. Differential usage of multiple brain-derived neurotrophic factor promoters in the rat brain following neuronal activation. Proc Natl Acad Sci U S A. 1993;90:8802–6. 8415610 10.1073/pnas.90.19.8802 PMC47448

[CR58] Molendijk ML, et al. Serum BDNF concentrations as peripheral manifestations of depression: evidence from a systematic review and meta-analyses on 179 associations (N=9484). Mol Psychiatry. 2014;19:791–800. 23958957 10.1038/mp.2013.105

[CR59] Mousavi K, Jasmin BJ. BDNF is expressed in skeletal muscle satellite cells and inhibits myogenic differentiation. J Neurosci. 2006;26:5739–49. 16723531 10.1523/JNEUROSCI.5398-05.2006 PMC6675269

[CR60] Murphy MC, Fox EA. Mice deficient in brain-derived neurotrophic factor have altered development of gastric vagal sensory innervation. J Comp Neurol. 2010;518:2934–51. 20533354 10.1002/cne.22372 PMC2888090

[CR61] Nakahashi T, et al. Vascular endothelial cells synthesize and secrete brain-derived neurotrophic factor. FEBS Lett. 2000;470:113–7. 10734218 10.1016/s0014-5793(00)01302-8

[CR56] National Library of Medicine (updated 2024) Bdnf brain derived neurotrophic factor [Mus musculus (house mouse)]. https://www.ncbi.nlm.nih.gov/gene/12064.

[CR62] Neeper SA, Gomez-Pinilla F, Choi J, Cotman C. Exercise and brain neurotrophins. Nature. 1995;373:109. 7816089 10.1038/373109a0

[CR63] Park H, Poo MM. Neurotrophin regulation of neural circuit development and function. Nat Rev Neurosci. 2013;14:7–23. 23254191 10.1038/nrn3379

[CR64] Phillips HS, et al. BDNF mRNA is decreased in the hippocampus of individuals with Alzheimer’s disease. Neuron. 1991;7:695–702. 1742020 10.1016/0896-6273(91)90273-3

[CR65] Pius-Sadowska E, Machalinski B. BDNF - A key player in cardiovascular system. J Mol Cell Cardiol. 2017;110:54–60. 28736262 10.1016/j.yjmcc.2017.07.007

[CR66] Preker P, et al. RNA exosome depletion reveals transcription upstream of active human promoters. Science. 2008;322:1851–4. 19056938 10.1126/science.1164096

[CR67] Pruunsild P, Kazantseva A, Aid T, Palm K, Timmusk T. Dissecting the human BDNF locus: bidirectional transcription, complex splicing, and multiple promoters. Genomics. 2007;90:397–406. 17629449 10.1016/j.ygeno.2007.05.004 PMC2568880

[CR68] Radka SF, Holst PA, Fritsche M, Altar CA. Presence of brain-derived neurotrophic factor in brain and human and rat but not mouse serum detected by a sensitive and specific immunoassay. Brain Res. 1996;709:122–301. 8869564 10.1016/0006-8993(95)01321-0

[CR69] Renteria I, et al. The molecular effects of BDNF synthesis on skeletal muscle: a mini-review. Front Physiol. 2022;13: 934714. 35874524 10.3389/fphys.2022.934714 PMC9306488

[CR105] Rohart F, Gautier B, Singh A, Lê Cao KA. mixOmics: An R package for ‘omics feature selection and multiple data integration. PLoS Comput Biol. 2017;13:e1005752 10.1371/journal.pcbi.1005752 PMC5687754 29099853

[CR70] Rosenfeld RD, et al. Purification and identification of brain-derived neurotrophic factor from human serum. Protein Expr Purif. 1995;6:465–71. 8527932 10.1006/prep.1995.1062

[CR71] Roth TL, Lubin FD, Funk AJ, Sweatt JD. Lasting epigenetic influence of early-life adversity on the BDNF gene. Biol Psychiatry. 2009;65:760–9. 19150054 10.1016/j.biopsych.2008.11.028 PMC3056389

[CR72] Sakata K, Duke SM. Lack of BDNF expression through promoter IV disturbs expression of monoamine genes in the frontal cortex and hippocampus. Neuroscience. 2014;260:265–75. 24345476 10.1016/j.neuroscience.2013.12.013

[CR73] Sakata K, Overacre AE. Promoter IV-BDNF deficiency disturbs cholinergic gene expression of CHRNA5, CHRM2, and CHRM5: effects of drug and environmental treatments. J Neurochem. 2017;143:49–64. 28722769 10.1111/jnc.14129 PMC5672805

[CR74] Sakata K, et al. Critical role of promoter IV-driven BDNF transcription in GABAergic transmission and synaptic plasticity in the prefrontal cortex. Proc Natl Acad Sci U S A. 2009;106:5942–7. 19293383 10.1073/pnas.0811431106 PMC2667049

[CR75] Sakata K, Jin L, Jha S. Lack of promoter IV-driven BDNF transcription results in depression-like behavior. Genes Brain Behav. 2010;9:712–21. 20528954 10.1111/j.1601-183X.2010.00605.x

[CR76] Sakata K, et al. Role of activity-dependent BDNF expression in hippocampal-prefrontal cortical regulation of behavioral perseverance. Proc Natl Acad Sci U S A. 2013a;110:15103–8. 23980178 10.1073/pnas.1222872110 PMC3773762

[CR77] Sakata K, et al. Effects of antidepressant treatment on mice lacking brain-derived neurotrophic factor expression through promoter IV. Eur J Neurosci. 2013b;37:1863–74. 23406189 10.1111/ejn.12148

[CR78] Schmidt HD, Duman RS. Peripheral BDNF produces antidepressant-like effects in cellular and behavioral models. Neuropsychopharmacology. 2010;35:2378–91. 20686454 10.1038/npp.2010.114 PMC2955759

[CR79] Sen S, Duman R, Sanacora G. Serum brain-derived neurotrophic factor, depression, and antidepressant medications: meta-analyses and implications. Biol Psychiatry. 2008;64:527–32. 18571629 10.1016/j.biopsych.2008.05.005 PMC2597158

[CR80] Sen A, Nelson TJ, Alkon DL. ApoE4 and Abeta oligomers reduce BDNF expression via HDAC nuclear translocation. J Neurosci. 2015;35:7538–51. 25972179 10.1523/JNEUROSCI.0260-15.2015 PMC6705431

[CR81] Sharma GP, et al. Brain-derived neurotrophic factor promotes immune reconstitution following radiation injury via activation of bone marrow mesenchymal stem cells. PLoS ONE. 2021;16: e0259042. 34695155 10.1371/journal.pone.0259042 PMC8544859

[CR82] Shieh PB, Hu SC, Bobb K, Timmusk T, Ghosh A. Identification of a signaling pathway involved in calcium regulation of BDNF expression. Neuron. 1998;20:727–40. 9581764 10.1016/s0896-6273(00)81011-9

[CR83] Sohrabji F, Miranda RC, Toran-Allerand CD. Identification of a putative estrogen response element in the gene encoding brain-derived neurotrophic factor. Proc Natl Acad Sci U S A. 1995;92:11110–4. 7479947 10.1073/pnas.92.24.11110 PMC40581

[CR84] Szuhany KL, Bugatti M, Otto MW. A meta-analytic review of the effects of exercise on brain-derived neurotrophic factor. J Psychiatr Res. 2015;60:56–64. 25455510 10.1016/j.jpsychires.2014.10.003 PMC4314337

[CR85] Tao X, Finkbeiner S, Arnold DB, Shaywitz AJ, Greenberg ME. Ca2+ influx regulates BDNF transcription by a CREB family transcription factor-dependent mechanism. Neuron. 1998;20:709–26. 9581763 10.1016/s0896-6273(00)81010-7

[CR86] Thoenen H. Neurotrophins and neuronal plasticity. Science. 1995;270:593–8. 7570017 10.1126/science.270.5236.593

[CR87] Tsankova NM, et al. Sustained hippocampal chromatin regulation in a mouse model of depression and antidepressant action. Nat Neurosci. 2006;9:519–25. 16501568 10.1038/nn1659

[CR88] Wan R, et al. Evidence that BDNF regulates heart rate by a mechanism involving increased brainstem parasympathetic neuron excitability. J Neurochem. 2014;129:573–80. 24475741 10.1111/jnc.12656 PMC4137462

[CR89] Wang N, Tian B. Brain-derived neurotrophic factor in autoimmune inflammatory diseases (Review). Exp Ther Med. 2021;22:1292. 34630647 10.3892/etm.2021.10727 PMC8461510

[CR90] Wang T, Xie K, Lu B. Neurotrophins promote maturation of developing neuromuscular synapses. J Neurosci. 1995;15:4796–805. 7623111 10.1523/JNEUROSCI.15-07-04796.1995 PMC6577890

[CR91] Wang T, et al. Effect of exercise training on the FNDC5/BDNF pathway in spontaneously hypertensive rats. Physiol Rep. 2019;7: e14323. 31883222 10.14814/phy2.14323 PMC6934876

[CR92] Want A, Morgan JE, Barde YA. Brain-derived neurotrophic factor measurements in mouse serum and plasma using a sensitive and specific enzyme-linked immunosorbent assay. Sci Rep. 2023;13:7740. 37173369 10.1038/s41598-023-34262-0 PMC10182034

[CR93] Weickert CS, et al. Reduced brain-derived neurotrophic factor in prefrontal cortex of patients with schizophrenia. Mol Psychiatry. 2003;8:592–610. 12851636 10.1038/sj.mp.4001308

[CR94] Wong J, et al. Promoter specific alterations of brain-derived neurotrophic factor mRNA in schizophrenia. Neuroscience. 2010;169:1071–84. 20553817 10.1016/j.neuroscience.2010.05.037 PMC3118308

[CR95] Wu D, Pardridge WM. Neuroprotection with noninvasive neurotrophin delivery to the brain. Proc Natl Acad Sci U S A. 1999;96:254–9. 9874805 10.1073/pnas.96.1.254 PMC15126

[CR96] Xiao R, et al. Enriched environment regulates thymocyte development and alleviates experimental autoimmune encephalomyelitis in mice. Brain Behav Immun. 2019;75:137–48. 30287389 10.1016/j.bbi.2018.09.028 PMC6279528

[CR97] Yamamoto H, Gurney ME. Human platelets contain brain-derived neurotrophic factor. J Neurosci. 1990;10:3469–78. 2230938 10.1523/JNEUROSCI.10-11-03469.1990 PMC6570101

[CR98] Yan Q, Elliott J, Snider WD. Brain-derived neurotrophic factor rescues spinal motor neurons from axotomy-induced cell death. Nature. 1992;360:753–5. 1281520 10.1038/360753a0

[CR99] Yang B, Ren Q, Zhang JC, Chen QX, Hashimoto K. Altered expression of BDNF, BDNF pro-peptide and their precursor proBDNF in brain and liver tissues from psychiatric disorders: rethinking the brain-liver axis. Transl Psychiatry. 2017;7: e1128. 28509900 10.1038/tp.2017.95 PMC5534963

[CR100] Yang X, et al. Myocardial brain-derived neurotrophic factor regulates cardiac bioenergetics through the transcription factor Yin Yang 1. Cardiovasc Res. 2023;119:571–86. 35704040 10.1093/cvr/cvac096 PMC10226756

[CR101] Yasutake C, Kuroda K, Yanagawa T, Okamura T, Yoneda H. Serum BDNF, TNF-alpha and IL-1beta levels in dementia patients: comparison between Alzheimer’s disease and vascular dementia. Eur Arch Psychiatry Clin Neurosci. 2006;256:402–6. 16783499 10.1007/s00406-006-0652-8

[CR102] Yu YB, et al. Brain-derived neurotrophic factor contributes to abdominal pain in irritable bowel syndrome. Gut. 2012;61:685–94. 21997550 10.1136/gutjnl-2011-300265

[CR103] Zhang Y, Qin G, Liu DR, Wang Y, Yao SK. Increased expression of brain-derived neurotrophic factor is correlated with visceral hypersensitivity in patients with diarrhea-predominant irritable bowel syndrome. World J Gastroenterol. 2019;25:269–81. 30670915 10.3748/wjg.v25.i2.269 PMC6337018

[CR104] Zhao DY, et al. Brain-derived neurotrophic factor modulates intestinal barrier by inhibiting intestinal epithelial cells apoptosis in mice. Physiol Res. 2018;67:475–85. 29527912 10.33549/physiolres.933641

