# Correction: Peripheral tissue BDNF expression is affected by promoter IV defect and enriched environments in mice: negative hippocampus-intestine and positive thymus-serum-muscle correlations

**DOI:** 10.1186/s10020-025-01317-z

**Published:** 2025-09-08

**Authors:** Janet Wang, William Schupp, Kazuko Sakata

**Affiliations:** 1 Department of Pharmacology, College of Medicine, University of Tennessee Health Science Center, 71 S. Manassas St. Room 225N, Memphis, TN 38103 USA; 2 Department of Psychiatry, College of Medicine, University of Tennessee Health Science Center, Memphis, USA

**Correction: Molecular Medicine 31, 164 (2025)**

**https://doi.org/10.1186/s10020-025-01196-4**

In Fig. 7 of this article (Wang et al. 2025); the panel [d] appeared incorrect. The explanation for the colored circles were missed and have now been corrected in the original publication. For completeness and transparency, both correct and incorrect versions are displayed below.

Incorrect version

**Fig. 7:**
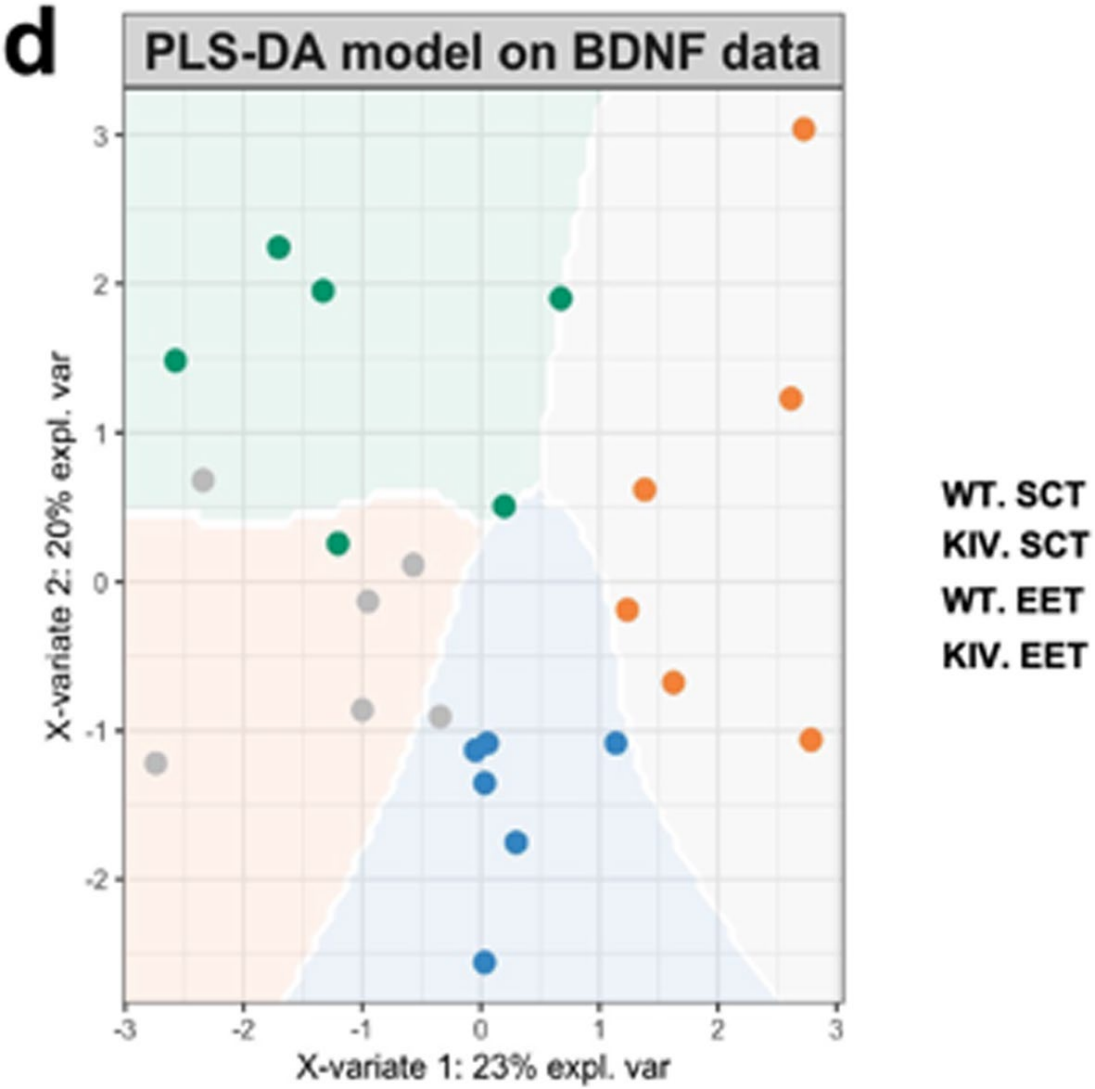
Partial Least Squares (PLS) regression model on tissue BDNF correlations to predict promoter IV defect and environments. (a) Correlation circle plot from PLS regression analyses to indicate the most important variables. Variables far from the center contribute more to the top two PLS components (largest variance). Variables closer to the center have less influence on the variation in the dataset. Variables closer together with a small angle between their vectors are positively correlated, while those with a large angle or positioned oppositely are negatively correlated. (b) Clustered image map on BDNF correlations across tissues. Similarity values and correlations between variables across two dimensions were clustered with a complete Euclidean distance method. The same colors indicate correlation in a similar direction, whereas opposing colors (red vs blue) indicate an opposite relationship. (c) Network representation showing a similarity matrix (0.45 cutoff). (d) Partial Least Squares discrimination analyses (PLS-DA) for classification model creation and validation. A prediction background (by the Mahalanobis distance metric) shows the classes that would be assigned to novel data points given their values on the first two latent components. (e) Cross-validation to find the optimal number of components. (f) Variable Importance in Projection (VIP) scores (> 1) indicate variables that significantly contribute to group separation among genotypes, treatments, and sexes; higher scores indicate more contribution. (g) VIP scores for genotype predictions and environment predictions. *WT* wild-type; *KIV* knock-in BDNF promoter IV: *SCT* standard control treatment; *EET* enriched environment treatment; *Comp* component

Correct version

**Fig. 7:**
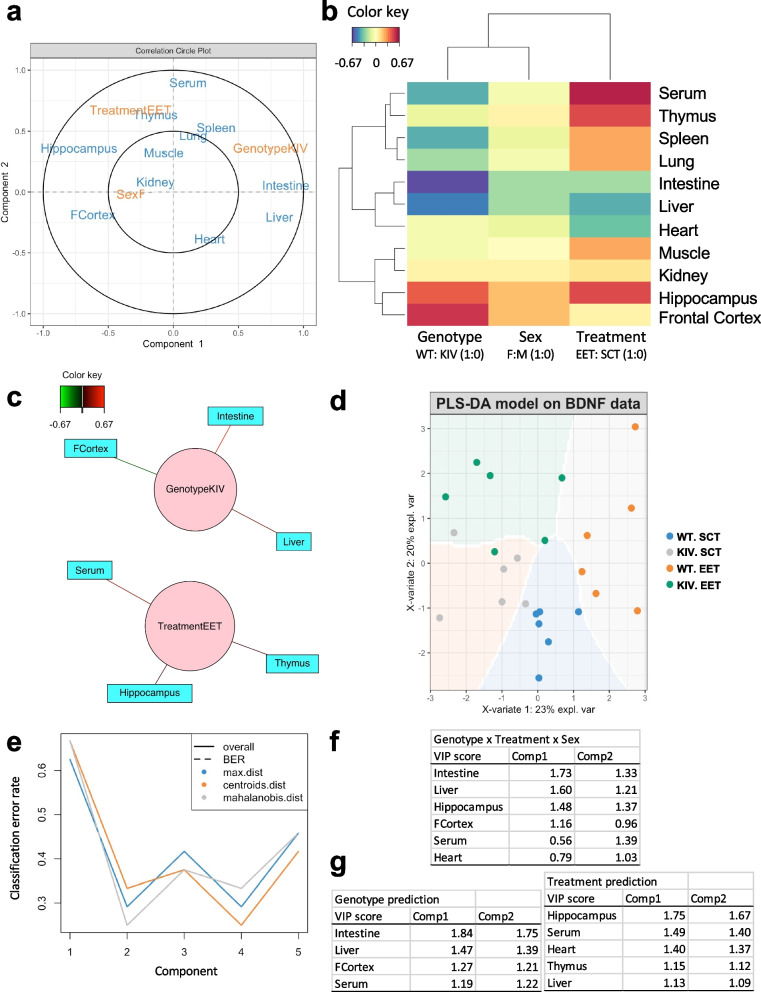
Partial Least Squares (PLS) regression model on tissue BDNF correlations to predict promoter IV defect and environments. (a) Correlation circle plot from PLS regression analyses to indicate the most important variables. Variables far from the center contribute more to the top two PLS components (largest variance). Variables closer to the center have less influence on the variation in the dataset. Variables closer together with a small angle between their vectors are positively correlated, while those with a large angle or positioned oppositely are negatively correlated. (b) Clustered image map on BDNF correlations across tissues. Similarity values and correlations between variables across two dimensions were clustered with a complete Euclidean distance method. The same colors indicate correlation in a similar direction, whereas opposing colors (red vs blue) indicate an opposite relationship. (c) Network representation showing a similarity matrix (0.45 cutoff). (d) Partial Least Squares discrimination analyses (PLS-DA) for classification model creation and validation. A prediction background (by the Mahalanobis distance metric) shows the classes that would be assigned to novel data points given their values on the first two latent components. (e) Cross-validation to find the optimal number of components. (f) Variable Importance in Projection (VIP) scores (> 1) indicate variables that significantly contribute to group separation among genotypes, treatments, and sexes; higher scores indicate more contribution. (g) VIP scores for genotype predictions and environment predictions. *WT* wild-type; *KIV* knock-in BDNF promoter IV: *SCT* standard control treatment; *EET* enriched environment treatment; *Comp* component

The original article has been corrected.
