# Supplementary material for: Peripheral tissue BDNF expression is affected by promoter IV defect and enriched environments in mice: negative hippocampus-intestine and positive thymus-serum-muscle correlations
Source: Mol Med. 2025 May 2;31:164. doi: 10.1186/s10020-025-01196-4 (PMC12048937; doi:10.1186/s10020-025-01196-4)
Supplement: Supplementary file 1 — Supplementary Fig. 1-6. [file 10020_2025_1196_MOESM1_ESM.pdf]

Supplementary Figure 1

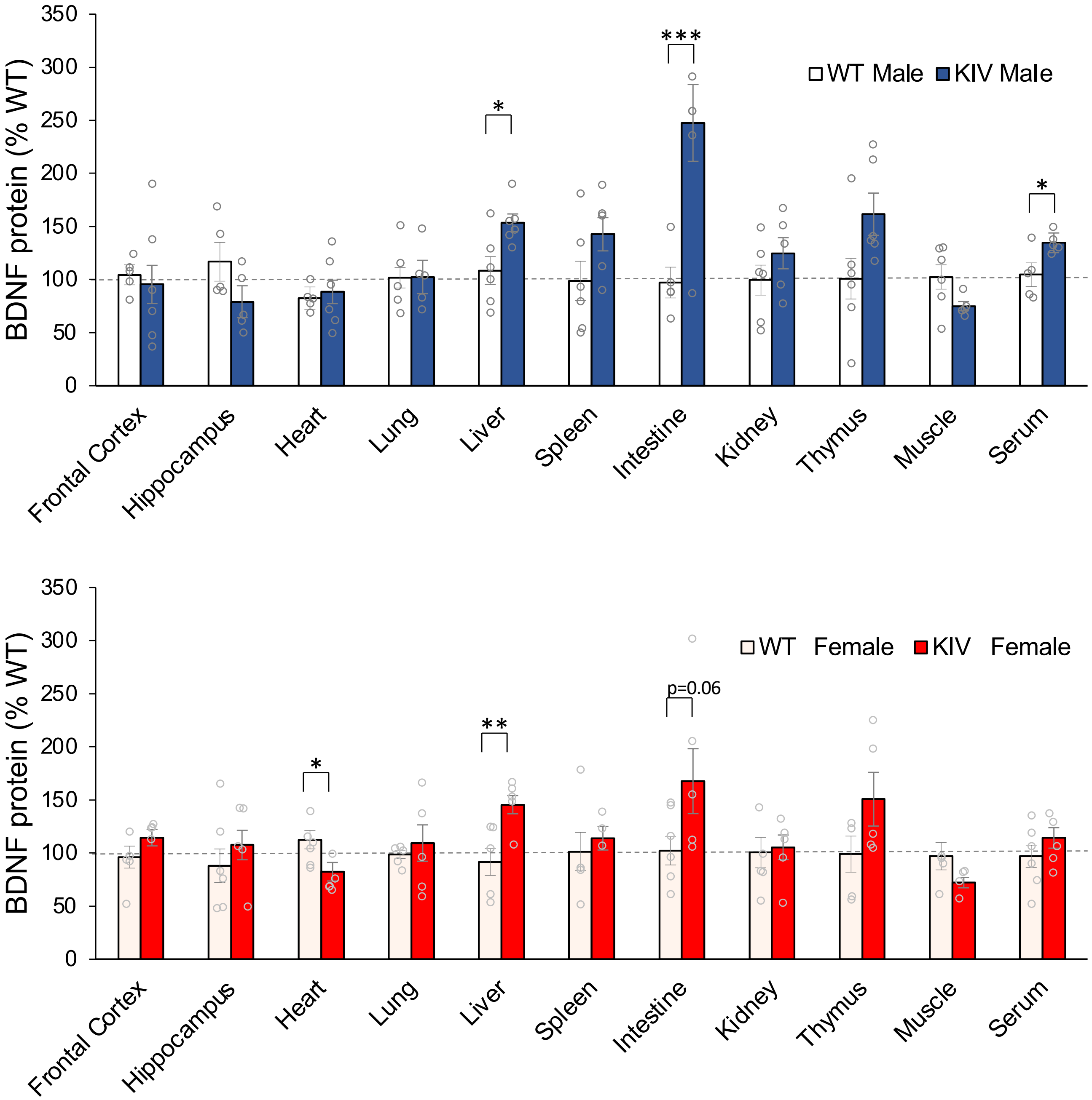

Supplementary Fig. 1. Sex effects on BDNF expression in standard control groups. Note that both males and females of promoter IV-BDNF deficient (KIV) mice showed similar pattern of BDNF expression levels across regions: significantly increased BDNF levels in the liver and intestine, a trend of increased BDNF levels in the thymus and serum and decreased BDNF levels in the muscle, when compared to WT mice. KIV female mice showed significantly decreased levels of BDNF in the heart. WT vs KIV: *$p<0.05$; **$p<0.01$; **$p<0.001$ by Student *t*-test. N=6 mice per group. BDNF levels (pg/mg total protein) were normalized to the mean of BDNF levels of each tissue of WT mice (% WT) and are shown as means ± S.E..

Supplementary Figure 2

a

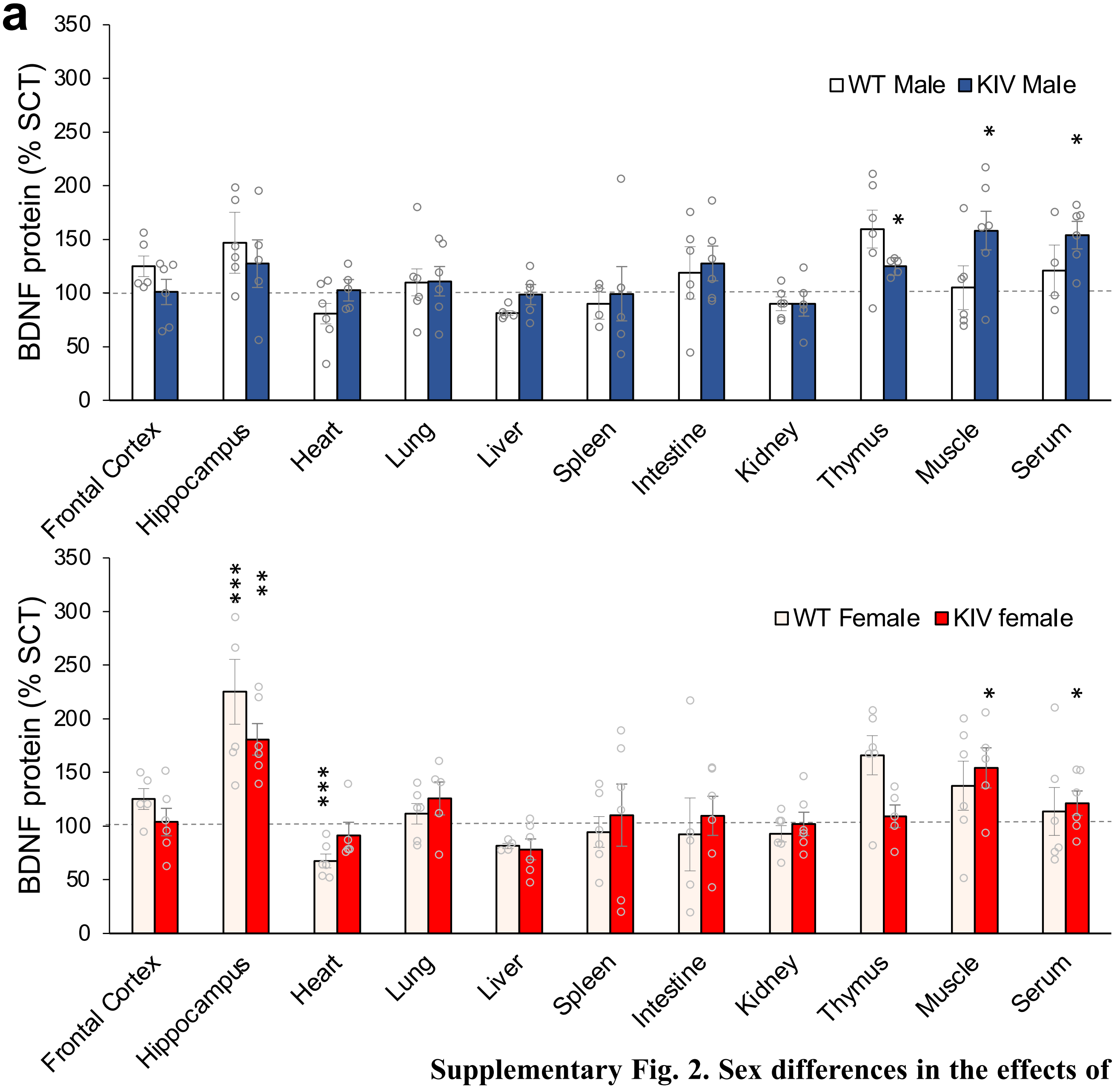

b

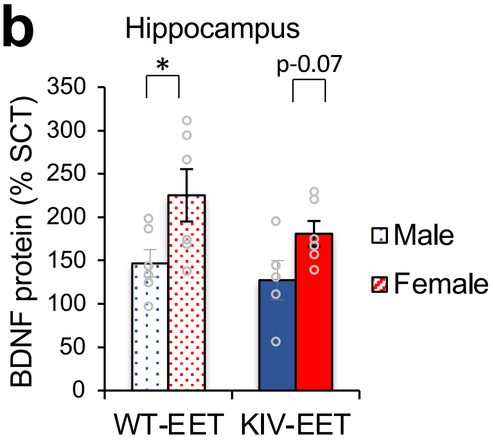

Supplementary Fig. 2. Sex differences in the effects of enriched environment treatment (EET) on BDNF. a. Males and females of both genotypes showed similar EET-induced changes in BDNF protein levels (% standard condition treatment: SCT). SCT vs EET: *p<0.05; **p<0.01; ***p<0.001 by Student's t-test. N=6 mice. group. **b.** There were no significant sex differences across the tissues except in the hippocampus, where WT females showed much higher EET-induced BDNF increases than WT males. Males vs females: *p<0.05 by Student's t-test. N=6 mice/group. BDNF levels (pg/mg total protein) were normalized to the mean of BDNF levels of each genotype with SCT (Means ± S.E.).

Supplementary Figure 3

a **WT**

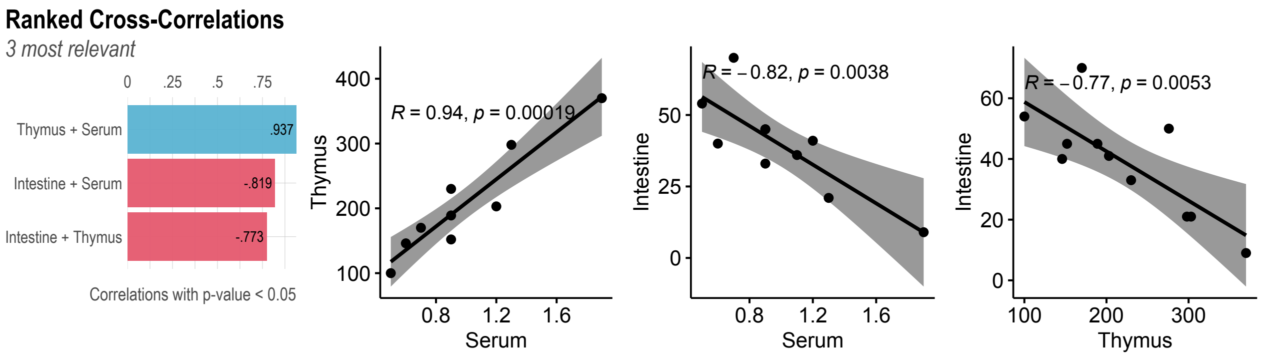

b **KIV**

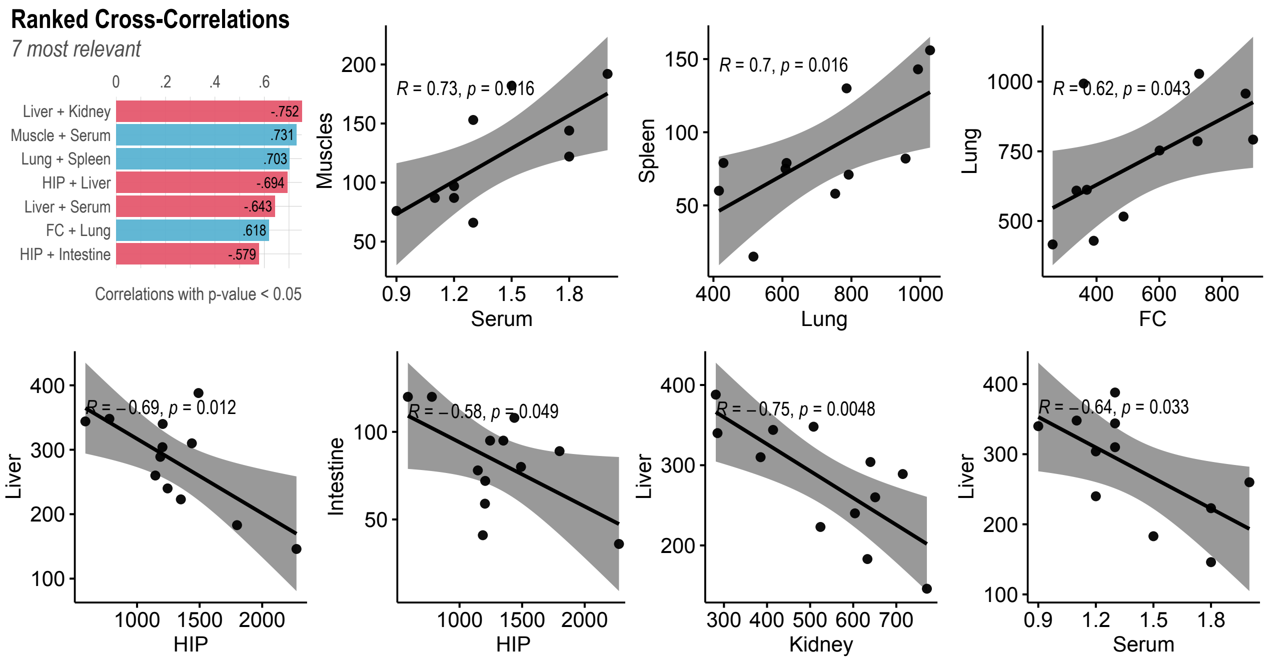

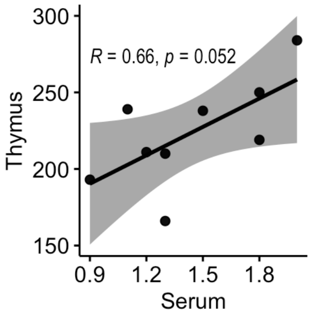

Supplementary Fig. 3. Pearson's ranked cross-correlation in (a) WT and (b) KIV mice from both SCT and EET groups. BDNF levels in pg per mg total protein are plotted. N=12 mice per group. Data from ELISA measuring BDNF levels of 11 regions on a same plate were used to avoid variations due to different ELISA plates and different experimental handling. HIP: hippocampus; FC: frontal cortex. KIV showed a trend of thymus-serum correlation (*p*=0.052).

Supplementary Figure 4

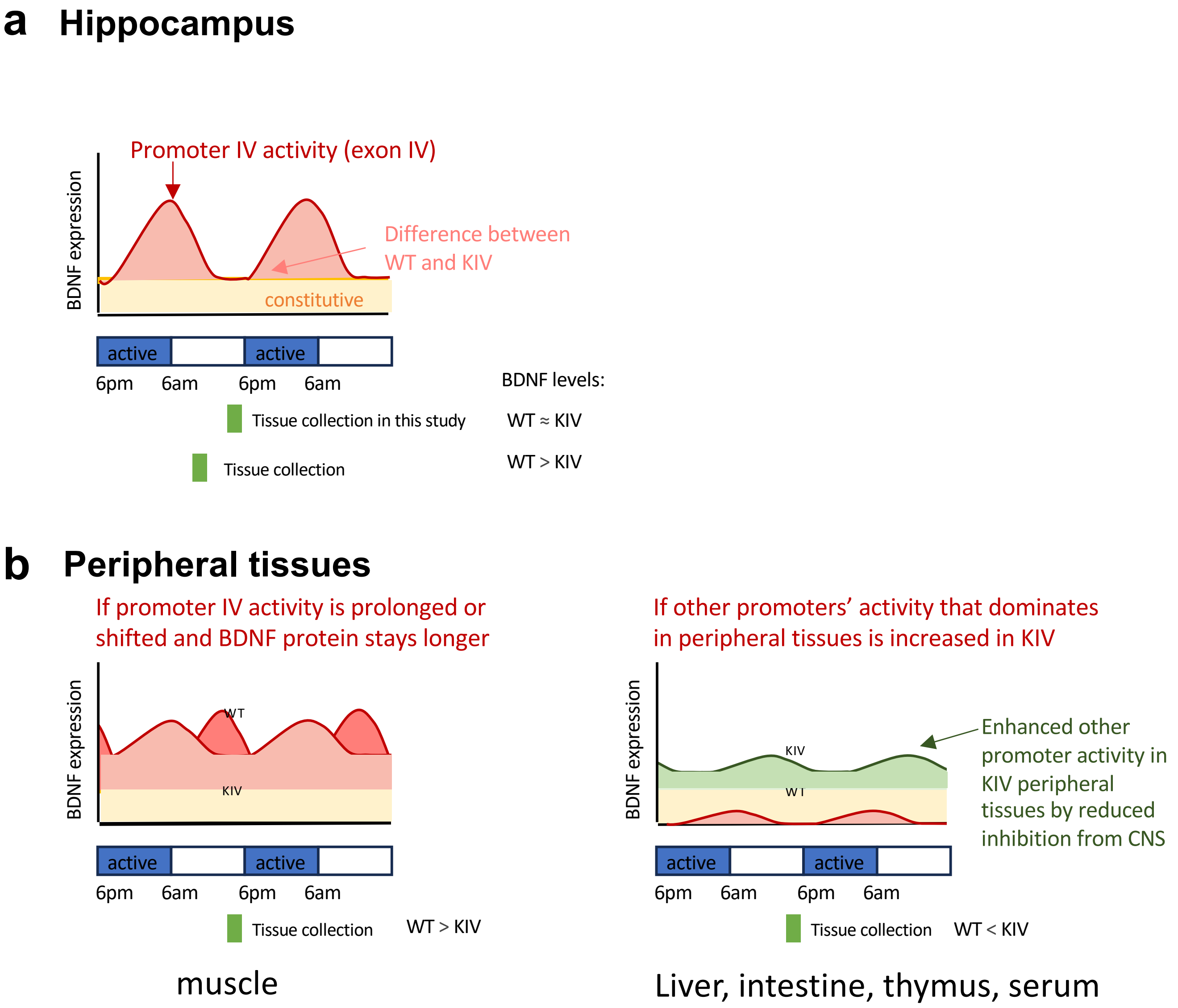

Supplementary Fig. 4. Possible model that explains why BDNF protein expression changed more in peripheral organs than in the central nervous system (CNS) in KIV mice. a. Promoter IV activity in the hippocampus shows diurnal change, which increases in the dark phase (active phase for nocturnal mice) and decreases in the light phase, being lowest before the next dark phase [38]. Tissues were collected in the late light phase before the dark phase started (~16:30 in this study). This was to examine chronic effects of enriched environmental treatment (EET, 3 weeks) on basal BDNF levels without the acute BDNF changes caused by individuals' daily activity (e.g., running). **b. Left:** In peripheral tissues, BDNF promoter IV activity can be prolonged or shifted (active during animals' sleep), while BDNF protein levels may persist without active release or degradation, unlike BDNF in the CNS which works as a neurotransmitter/modulator. **Right:** Other promoter activity in peripheral tissues may be enhanced due to reduced neuronal inhibition caused by reduced activity-induced BDNF levels in the CNS.

Supplementary Figure 5

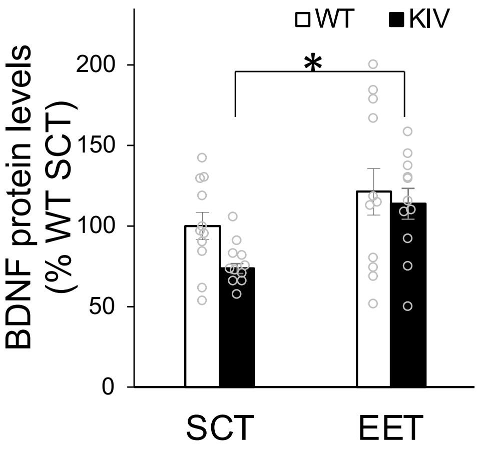

Supplementary Fig. 5. BDNF levels in muscle. KIV mice showed significantly reduced BDNF levels compared to WT mice, while EET increased (KIV-SCT versus KIV-EET, *$p<0.05$ by Bonferroni's comparison) and normalized (WT EET versus KIV EET $p>0.05$ by Student *t*-test). $F_{treatment} = 8.9$, $p<0.01$ by Two-way ANOVA. N=12 mice per group.

Supplementary Figure 6

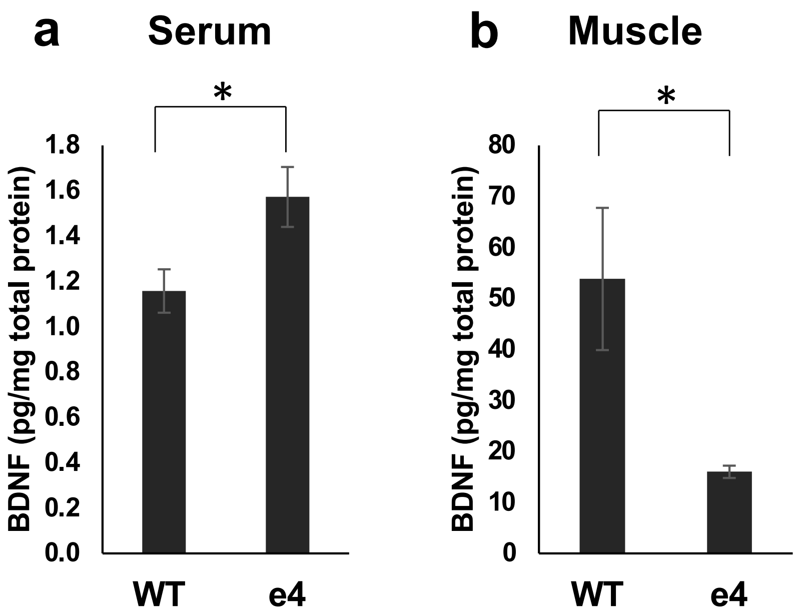

Supplementary Fig. 6. BDNF levels in serum and muscle of a second-generation line of KIV (e4) mice that lack the PGK promoter-Neo cassette. A significant increase in serum BDNF levels (a) and a significant reduction in muscle BDNF levels (b) were observed in e4 mice [104], reproducing the results of KIV mice (**Fig. 2**). Thus, it is unlikely that the PGK (phosphoglycerate kinase) promoter in the Neo selection cassette acts as a cryptic promoter, transcribing its upstream BDNF protein-coding exon. KIV mice contain PGK promoter in exon IV in the antisense direction of exon IV [21]. PGK promoter is a primary unidirectional (5' to 3') with limited 3'to 5' transcription activity that would terminate within ~2.5 kb transcription [102][103]. However, the PGK-Neo cassette may cause artifacts such as DNA structural change to suppress or enhance BDNF transcription in peripheral tissues. Therefore, the effects were tested. WT mice versus e4 mice: *$p<0.05$ by Student-*t* test. N=4 mice per group.
